# Behavioral Signatures of Post-Decisional Attention in Preferential Choice

**DOI:** 10.64898/2026.01.10.698805

**Published:** 2026-01-12

**Authors:** Ariel Zylberberg, Ian Krajbich, Michael N. Shadlen

**Affiliations:** 1 Mortimer B Zuckerman Mind Brain Behavior Institute, Columbia University, New York, United States; 2 Virtual Confidence and Metacognition Laboratory; 3 University of California Los Angeles, Department of Psychology, Los Angeles, CA, USA; 4 Department of Neuroscience, Columbia University, New York, United States; 5 The Kavli Institute for Brain Science, Columbia University, New York, United States; 6 Howard Hughes Medical Institute, Chevy Chase, United States

## Abstract

Attention plays a key role in decision-making by directing limited cognitive resources to relevant information. It has been proposed that attention also biases the decision process, due to a multiplicative interaction between attention and subjective value (e.g., Krajbich et al., 2010). We tested two predictions of models that posit a causal multiplicative effect of attention on decision formation: (i) the last fixation should be more informative about the choice when the overall value of the alternatives is high, and (ii) more attention should be directed to the chosen option when choices conflict with stated preferences than when they do not. Reanalyzing data from a food-choice task (Krajbich et al., 2010), we found no evidence supporting these predictions. A similar discrepancy with the data is observed in recent normative models, which propose that gaze allocation arises from a process of Bayesian inference about the latent values of the alternatives (Callaway et al., 2021; Jang et al., 2021). An alternative model where attention reflects choices after the decision has completed, explains key observations, including the last-fixation bias, the gaze-cascade effect and the effect of the overall value of the alternatives on response times. However, this model does not fully account for the association between dwell time and choice. We conclude that gaze behavior prior to the choice report likely reflects both decisional and post-decisional processes.

## Introduction

Attention plays a key role in decision making, enabling individuals to focus on relevant information while ignoring distractions. It has also been hypothesized that attention biases decision makers’ preferences and choices. During decisions involving spatially distributed stimuli, gaze progressively shifts toward the option ultimately chosen, a phenomenon termed the gaze cascade effect (Shimojo et al., 2003; Glaholt and Reingold, 2009). Additionally, decision-makers often select the option they fixate on last before reporting their choice (a “last-fixation” bias; Krajbich et al. 2010). Experimental manipulations of gaze, including spatial cues, variations in exposure duration, and salience control, have demonstrated that options receiving more attention are more likely to be chosen (Nittono and Wada, 2009; Bhatnagar and Orquin, 2022; Pleskac et al., 2023). As gaze reflects spatial attention, these findings support the idea that attention biases choice (Shimojo et al., 2003; Armel et al., 2008; Zajonc, 1968).

The attentional drift diffusion model (*aDDM*) formalizes this hypothesis in a way that makes it suitable to quantitatively explain choice and response time (RT) (Krajbich et al., 2010). The *aDDM* builds on the drift-diffusion model of decision making (*DDM*), in which decisions are made by accumulating noisy samples of momentary evidence over time (Ratcliff, 1978). In preference-based decisions, the momentary evidence depends on the subjective value difference between the options. The decision is thought to terminate when the accumulated evidence crosses an upper or lower bound, simultaneously resolving the choice and the time it took to make it. The *DDM* and some of its variants, such as random walk, race, and attractor models, have been successful in explaining both choice and RT in a range of perceptual and cognitive decisions in which no role for attention is assumed (Link, 1975; Ratcliff and McKoon, 2008; Usher and McClelland, 2001; Vickers, 1979; Wang, 2002).

The *aDDM* extends the *DDM* by including a role for attention in the decision process. It proposes that attention can change the subjective value of decision alternatives: specifically, the value of unattended options is discounted by a multiplicative factor. This framework assumes that attention exerts its influence **intra-decisionally**—that is, during the deliberation process, before a choice is made. Originally developed for preference-based decisions between two options, the *aDDM* has been extended to multialternative (Krajbich and Rangel, 2011; Thomas et al., 2021), perceptual (Smith and Krajbich, 2019; Tavares et al., 2017), purchase (Krajbich et al., 2012), and attribute-based decisions (Fisher, 2021; Yang and Krajbich, 2023). The model provides an explanation for the association between choice, RT, subjective value, and gaze allocation, including the apparent causal influence of gaze on choice (for a review see Krajbich, 2019).

The allocation of attention in the *aDDM* is exogenous to the decision process—that is, attention shifts between items independently of the internal dynamics of decision making. In contrast, more recent studies model the control of attention as endogenous, arising from an optimization that balances the cost of delaying the decision and collecting additional evidence, with the expected benefit of improved accuracy. For instance, Callaway et al. (2021) formalized the decision process as a partially observable Markov decision process (POMDP), and approximated its solution by assuming that the value of additional deliberation can be expressed as a linear combination of factors such as the expected reward from acquiring an extra evidence sample and the expected reward if the item values were perfectly known. They fit this model jointly to datasets involving two- and three-alternative choices (from Krajbich et al. 2010 and Krajbich and Rangel 2011, respectively), capturing several aspects of the data.

A key assumption in Callaway’s model is that the mean of the prior probability over the value of the items is lower than the true mean. This assumption leads to a tendency to choose the most frequently sampled item, since the bias in the prior affects the least frequently sampled option more strongly. Jang et al. (2021) derived an optimal decision policy based on a similar assumption. Because they focused on two-alternative choices, they were able to solve the Bellman equations by backward induction without resorting to the kind of approximations required by Callaway et al. (2021).

Here we explore two novel behavioral predictions of models that posit a multiplicative effect of attention on value. While past analyses showed an overall-value dependent influence of dwell time on choice (Smith and Krajbich, 2019), our new predictions concern (*i*) how the link between last fixation and choice depends on the overall value of the alternatives, and (*ii*) how the difference in dwell time between items varies with the consistency between choices and the initial value ratings. We tested these predictions by reanalyzing data from previously published value-based decision tasks (Krajbich et al., 2010; Smith and Krajbich, 2018; Chen and Krajbich, 2016; Gwinn, 2016; Folke et al., 2016; Sepulveda et al., 2020). Our results reveal systematic deviations from the predictions of both the *aDDM* and the optimal inference models proposed by Callaway et al. (2021) and Jang et al. (2021). Instead, the data suggest that the association between gaze and choice is partially **post-decisional**—emerging after a covert commitment to a decision but before the overt response is made (Cavanagh et al., 2014; Westbrook et al., 2020).

## Results

**Figure 1.**
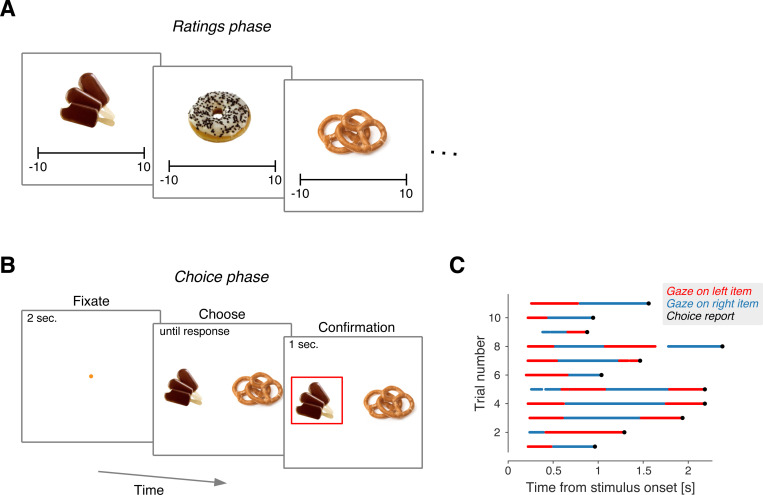
Food-choice task The task consisted of two phases. In the first phase (A), participants were shown 70 pictures of snack items, one at a time, and were asked to rate how much they would like to consume each item on a scale from −10 to 10. In the second phase (B), participants were presented with pairs of items that had received non-negative ratings in the first phase and were asked to choose which item they preferred. Before the snack items were presented, participants had to fixate on a central marker for 2 seconds. To report their choice, participants used the left and right arrows on a keyboard. After the choice report, the items were displayed for an additional second, during which time the selected item was highlighted. Each of the 39 participants completed 100 trials. (C) Gaze allocation between the left and right items shown from the moment both snacks appeared on the screen, for 11 representative trials. Red and blue indicate gaze directed to the left and right items, respectively. Times when the gaze was not directed to either item are left blank. Black dots indicate the time when the left or right key was pressed.

In the experiment of Krajbich et al. (2010), 39 hungry participants made decisions about snack items. The experiment consisted of two stages. In the first stage, participants viewed 70 items and rated how much they would like to consume each one on a numerical scale from –10 to 10 (Fig. 1A). In the second stage, participants were presented with pairs of items with non-negative ratings and asked to choose which one they would prefer to consume at the end of the experiment (Fig. 1B). Krajbich et al. (2010) tracked the participants’ gaze as they made the choices and identified the moments when the gaze was directed to the left and right items (Fig. 1C).

### The attentional drift-diffusion model

The decision-making process in the *aDDM* is governed by the state of a scalar decision variable, x, which takes the value zero at the start of each trial. The decision variable is updated according to

(1) $$x_{t}=x_{t-1}+d\left(r_{\text{left}}-\theta r_{\text{right}}\right)+\epsilon_{t}$$

when the decision maker is looking at the left item, and according to

(2) $$x_{t}=x_{t-1}+d\left(\theta r_{\text{left}}-r_{\text{right}}\right)+\epsilon_{t}$$

when looking at the right item. $r_{\text{left}}$ and $r_{\text{right}}$ represent the values assigned to the left and right items during the rating phase, respectively. If the decision variable reaches a bound at +1, the decision-making process terminates and the left item is selected, and if it reaches −1, the right item is selected. The parameter d controls the integration speed, θ is a parameter between 0 and 1 that determines how much the value of the unattended item is discounted, and $\epsilon_{t}$ is white Gaussian noise with variance $\sigma^{2}$. The difference between the *aDDM* and the standard drift-diffusion model is that gaze modulates the drift rate through the parameter θ.

### The last-fixation bias on choice does not increase with overall value

A central feature of the *aDDM* is the multiplicative interaction between gaze and value. This multiplicative interaction accounts for faster response times when the overall value of the options is high—a phenomenon we refer to as the *magnitude effect on response times* (MERT) (Smith and Krajbich, 2019; Ratcliff et al., 2018). Given that attention has a greater influence when both options are highly valued, the last-fixation bias—the tendency to choose the item that was fixated last—should also be amplified under these conditions. We refer to this novel prediction of the *aDDM* as the *magnitude effect on the last-fixation bias* (MELFB).

We illustrate the MELFB prediction through simulations of the *aDDM* using the best-fitting parameters identified by Krajbich et al. (2010) and Smith and Krajbich (2019). To evaluate the impact of attention on choice, we apply logistic regression with the following model:

(3) $$\text{logit}\left[p_{\text{left}}\right]=\sum_{s}\left(\beta_{\text{Δ}r,s}\text{Δ}r+\beta_{0,s}\right)I_{s}+\sum_{g}\beta_{\text{LastFix},g}I_{g}I_{\text{LastFix}},$$

where Δr represents the difference in value between the left and right items. $I_{s}$ is an indicator variable that equals 1 for trials completed by subject s and 0 otherwise. $I_{\text{LastFix}}$ is another indicator variable, set to 1 if the left item was fixated last and 0 if the right item was fixated last. We categorize trials into quintiles of Σr, the sum of the rating assigned to the left and right items; the variable $I_{g}$ identifies trials belonging to quintile g∈{1..5}. The β s are the regression coefficients.

The first set of terms on the right-hand side of the equation (the summation over s) captures the effect of Δr (along with a participant-specific bias) on the probability of choosing the left item. The second set of terms (the summation over g) reflects the influence of attention, measured by whether the left or right item was fixated last, with this effect estimated for each quintile of Σr.

**Figure 2.**
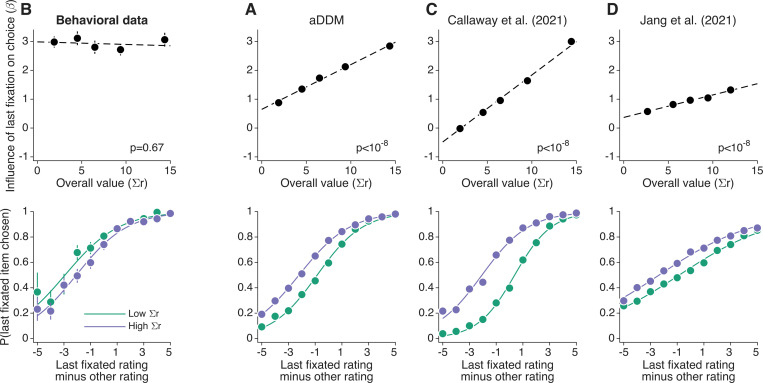
Magnitude effect on last-fixation bias (top) Influence on choice of the gaze right before the choice report, as measured with logistic regression (Eq. 3), as a function of the sum of the ratings assigned to of the left and right item (‘overall’ value or Σr). The five data points correspond to quintiles of the data, split by Σr. The dashed line is obtained from a related regression model that includes an interaction term between Σr and whether the last fixation was on the ultimately chosen item or on the unchosen item before the report. Error bars are s.e. The p-value indicated in each panel corresponds to a test of the hypothesis that the slope of the dashed line is equal to zero, evaluated using the z-test. (bottom) Proportion of trials for which participants (or the simulations) chose the item looked at last, as a function of the difference in value between the item looked at last and the other item. Trials were median-split by Σr. The choice functions were calculated per participant and then averaged across participants. Error bars are s.e.m. across participants. The four panels correspond to (A) simulations of the aDDM, (B) behavioral data from Krajbich et al. (2010), (C) Callaway et al’s model (2021), and (D) Jang et al’s model (2021).

As mentioned, the *aDDM* predicts that the effect of attention on choice increases with the total value of the items. Fig. 2A(top) shows the regression coefficient $\beta_{\text{LastFix,}g}$ for the different quintiles of overall value, for simulations of the *aDDM* model. The dashed black line is not a fit to these data points, but is obtained from a model similar to that of Eq. 3 except that the term associated with the quantiles of overall value is replaced by an interaction between $I_{\text{LastFix}}$ and Σr (Eq. 12). The analysis shows that the influence of the item attended last on choice increases with the overall value of the items (Fig. 2A, top).

A potentially more intuitive way of showing the effect of Σr as a modulator of the effect of attention on choice is shown in Fig. 2A(bottom). It shows the proportion of trials for which the participants chose the item they looked at last, as a function of the difference in rating between the item looked at last and the other item. The analysis was performed independently for trials with low and high Σr (relative to the median). For the simulations of the *aDDM*, the tendency to choose the last fixated item is lower when Σr is small than when Σr is large.

We tested these predictions of the *aDDM* with data from the food-choice task. We fit the logistic regression model (Eq. 3) to the data of Krajbich et al. (2010). No significant effect was observed for the effect of Σr on the strength of the association between gaze and choice (Fig. 2B, top). A similar conclusion is reached by analyzing the choice functions median-split by the Σr : the influence of the last fixation on choice is not stronger when Σr is high (Fig. 2B, bottom). That is, in contrast with model’s prediction, the last-fixation bias does not increase with the overall value of the alternatives.

### The difference in dwell time is independent of choice consistency

**Figure 3.**
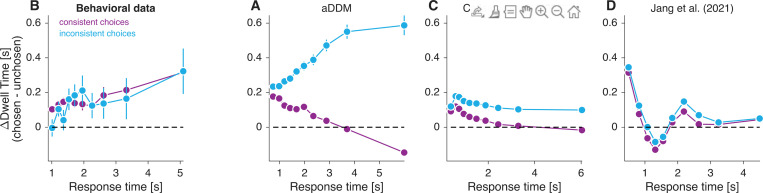
Difference in looking time for consistent and inconsistent choices Time spent looking at the chosen item minus time spent looking at the unchosen item, as a function of response time. The four panels correspond to (A) simulations of the *aDDM*, (B) behavioral data from Krajbich et al. (2010), (C) Callaway et al’s model, and (D) Jang et al’s model. For each participant, we grouped trials into 20 categories defined by the response time decile and the consistency of the choice with the initial rating. Trials in which the two items were assigned the same value during the rating phase were excluded from the analysis, as the choices cannot be classified neither as consistent nor inconsistent. The response times shown on the abscissa correspond to the mean response times (across participants) for the corresponding decile. Error bars are s.e.m. across participants.

People tend to look longer at the item they end up choosing than at the one they do not (Krajbich et al., 2010; Krajbich and Rangel, 2011). We refer to the difference in total fixation time between the chosen and unchosen items as ΔDwell. The *aDDM* predicts how ΔDwell should depend on the consistency of the choice with the item ratings. We define inconsistent choices as those in which the lower-rated item is selected over the higher-rated one. According to the *aDDM*, if individual dwells are not influenced by item value, then ΔDwell should be greater for inconsistent choices than for consistent ones. This prediction is confirmed in our simulations of the *aDDM* (Fig. 3A; p < 10^−7^, Eq. 13, $H_{0}:\beta_{c}=0$).

This prediction can be understood as follows. If, in a given trial, the decision maker had primarily attended to the higher-rated item, the likelihood of making an inconsistent choice would be lower than if attention had been equally divided between the options, because attention would have increased the extent to which the drift rate favored the higher-rated item. Thus, when a choice is known to be inconsistent, it is more likely that gaze was predominantly directed to the lower-rated item.

This logic also applies to consistent choices, but to a lesser extent. Even if attention is primarily allocated to the lower-valued item during a trial, it is still highly likely that the decision-maker will ultimately choose the higher-valued item. This is because, while attending to the lower-valued item reduces the effective drift rate in favor of the higher-valued item, it does not usually result in a change in the sign of the drift rate. Therefore, knowing that the decision was consistent does not provide as much information about the allocation of attention as knowing that the decision was inconsistent.

A similar logic explains why ΔDwell should depend on response time. The *aDDM* predicts that ΔDwell increases with RT for inconsistent choices (Fig. 3A). This is because the response time sets an upper limit on ΔDwell, so the longer the RT, the greater the value that ΔDwell can be. In contrast, ΔDwell decreases—and even becomes negative—with RT for consistent choices (Fig. 3A). This is because if the response time is longer than expected—given the values of the items being compared—then it is likely that attention was mostly focused on the lower-valued item. The effect is not as strong for inconsistent choices, because even if attention were primarily focused on the lower-valued item, the drift rate would still usually favor the unselected, higher-valued item.

We tested this prediction on the data from Krajbich et al. (2010). We found no significant differences in ΔDwell for consistent and inconsistent choices (Fig. 3B) (p = 0.12, Eq. 13, $H_{0}:\beta_{c}=0$). This is incompatible with the prediction of the *aDDM*. This incompatibility is not specific to the *aDDM*, but as we show next, applies to other instantiations of models that posit a causal influence of attention on choice, including those in which gaze is optimally allocated.

### Optimal models of decision making

We asked whether the optimal attentional allocation model of Callaway et al. (2021) would show the same discrepancies with the behavioral data as the *aDDM*. To this end, we reanalyzed the simulations of the optimal model performed by Callaway and colleagues. Like the *aDDM*, the model of Callaway et al. (2021) incorrectly predicts a strong effect of overall value on the strength of the association between attention and choice (Fig. 2C). This is clearly counter to what is observed in the data (Fig. 2B).

We also analyzed the association between ΔDwell and choice consistency. The results of this analysis are shown in Fig. 3C. The model of Callaway et al. (2021) predicts that ΔDwell depends on choice consistency, with greater ΔDwell for inconsistent choices. The explanation is the same as for the *aDDM*: if attention has a causal influence on choice, then for inconsistent choices it is highly likely that attention was directed to the item of lower value for a substantial fraction of the trial. Again, this is in clear contrast to what we observe in the data (Fig. 3B).

We also performed simulations of the ‘optimal’ model of Jang et al. (2021), using the best-fitting parameters reported in their study. The model predicts that ΔDwell changes sign as a function of response time (Fig. 3D), contrary to what is observed in the data (Fig. 3B). For example, at response times of ∼1.5 s, it incorrectly predicts that people will tend to choose the option they looked at the least. The model of Jang et al. (2021) also predicts that the influence of the last fixation on choice depends on Σr (Fig. 2D), contrary to what is observed in the data (Fig. 2B). We conclude that neither the *aDDM* nor the POMDP-based models account for the aspects of the data analyzed in Figs. 2 and 3.

### Is the gaze-choice association purely post-decisional?

The aspects of the data we analyzed in Figs. 2 and 3 are suggestive of a non-multiplicative interaction between attention and value (Cavanagh et al., 2014; Westbrook et al., 2020). We explore a model in which the link between gaze and choice arises only after a choice has been covertly made. Attention has no effect on the choice itself or on the time taken to make the choice. We refer to this model as the Post-Decision-Gaze (*PDG*) model.

In the *PDG* model, the decision is made by accumulating momentary evidence over time. The momentary evidence is represented by samples from a Gaussian distribution. The mean of the sampling distribution is a linear function of Δr, such that the drift rate of the drift-diffusion process (μ) is given by:

(4) $$\mu =\kappa \left(r_{\text{right}}-r_{\text{left}}\right),$$

where κ is a signal-to-noise parameter. Unlike the *aDDM*, the drift-rate is not modulated by the focus of attention.

Unlike most implementations of the DDM, in the *PDG* model the variance of the momentary evidence depends on Σr. This assumption is needed to explain why response times depend on both Δr and Σr (Ratcliff et al., 2018). We parameterize the variance as

(5) $$\sigma^{2}=1+\gamma \left(r_{\text{left}}+r_{\text{right}}\right),$$

where γ is fit to the data. The assumption has empirical support in neurobiology (Discussion).

**Figure 4.**
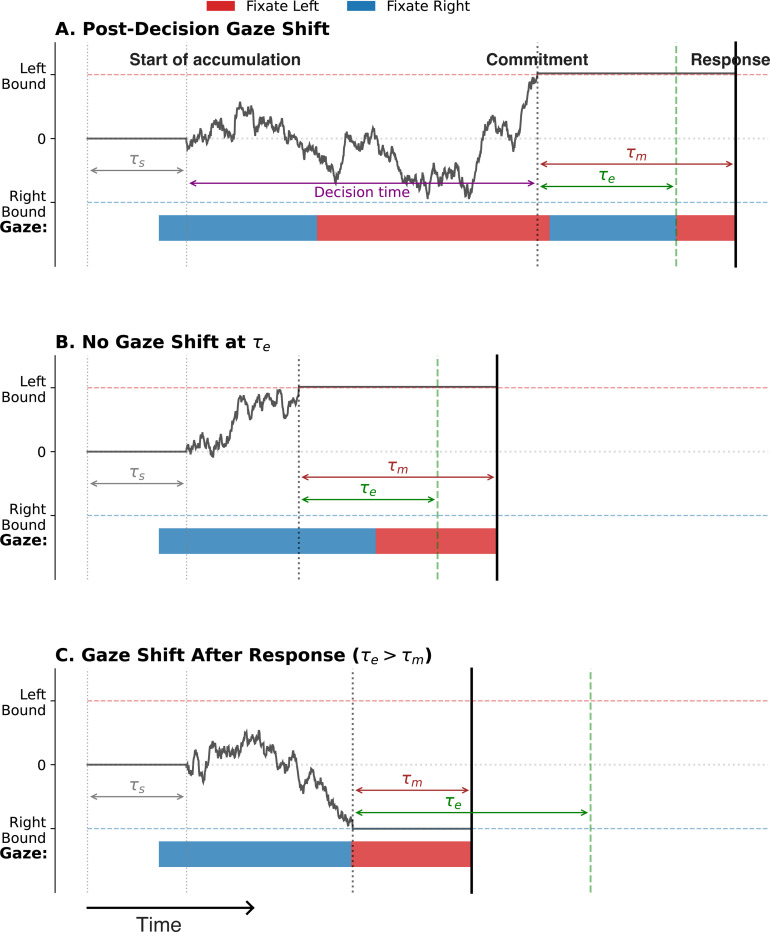
Sketch of the *PDG* model (A) The decision is generated by accumulating momentary evidence over time until the process reaches either an upper or lower bound. Evidence accumulation begins after a sensory delay, $\tau_{\text{s}}$. Once a bound is crossed, an additional motor delay, $\tau_{\text{m}}$, elapses before the manual response is executed. Thus, the response time equals the decision time plus the non-decision delays $\tau_{\text{s}}$ and $\tau_{\text{m}}$. Gaze does not affect the decision process. Instead, at a random latency *𝜏*_e_ after the bound crossing, the gaze is directed toward the chosen option and remains there until the manual response. Because $\tau_{\text{e}}$ is typically shorter than $\tau_{\text{m}}$, the chosen item is usually the last fixated item before the response. (B) Example simulation in which the gaze is already on the chosen item at time $\tau_{\text{e}}$ following bound crossing; therefore, no gaze shift occurs. (C) Simulation in which the gaze shift to the chosen item occurs only after the manual response, and therefore does not influence pre-response gaze behavior. This explains why, in some trials—including the example shown—the non-chosen item is the last one fixated before the response.

The evidence accumulation process begins after a short sensory delay, $\tau_{\text{s}}$ (Fig. 4A). For the purposes of our analysis, we fixed $\tau_{\text{s}}$ at 0.3 s for all participants. Note that while neurophysiological studies in monkeys have estimated that $\tau_{\text{s}}$ is on the order of 0.2 s (Roitman and Shadlen, 2002; Steinemann et al., 2022), $\tau_{\text{s}}$ is likely to be longer in the food-choice task since participants start each trial fixating on a central spot before directing their gaze to one of the choice alternatives (Krajbich et al., 2010).

The model also includes a non-decision latency, $\tau_{\text{m}}$, between the time when the decision maker commits to a choice, signaled by crossing the decision threshold, and the time when a key is pressed to report the choice (Fig. 4A). $\tau_{\text{m}}$ accounts for latencies related to motor preparation and is independent of decision difficulty. The response time is given by the sum of $\tau_{\text{m}}$, $\tau_{\text{s}}$, and the decision time (Fig. 4A).

In the model, attention has no causal effect on the decision process. Therefore, there is a 50% chance that the gaze will be directed to either item at the time a threshold is crossed. The key assumption of the model is that once the decision variable crosses the decision threshold, the gaze is directed to the chosen item. To account for eye-movement related latencies, we assume that directing gaze to the chosen item occurs with a latency of $\tau_{\text{e}}$ from the time of bound crossing (Fig. 4A), after which the gaze is held on the chosen item until the response. If the decision maker was already looking at the chosen item after $\tau_{\text{e}}$ has elapsed from the time of bound crossing, no additional gaze shift occurs (Fig. 4B).

Because the time it takes to make an eye movement is usually less than the time it takes to complete the manual response, $\tau_{\text{m}}$, the decision maker is more likely to be looking at the chosen item when a key is pressed (Fig. 4A,B). However, due to variability in $\tau_{\text{m}}$ and in $\tau_{\text{e}}$, the time at which gaze is directed to the chosen item after choice commitment varies from trial to trial, and may even occur after the key press, as in Fig. 4C. Crucially, in contrast with the *aDDM* and related models (Krajbich et al., 2010; Thomas et al., 2019; Krajbich and Rangel, 2011), it is the choice that affects gaze allocation, not the other way around.

### Fits of the *PDG* model to the behavioral data

**Figure 5.**
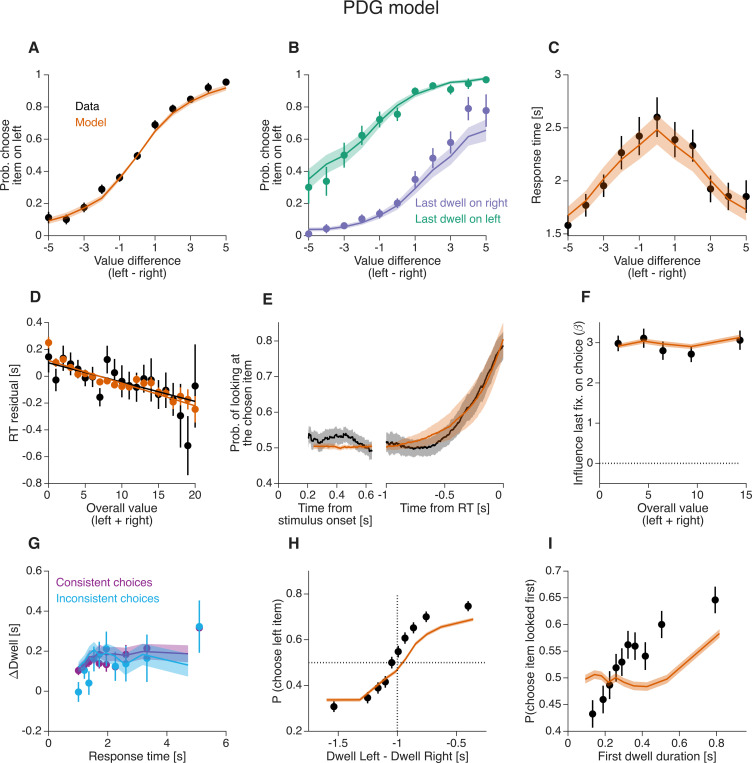
Human behavior and fits of the *PDG* model (A) Proportion of trials in which the right item was selected as a function of the difference in value between the right and left items $\left(\text{Δ}r\right)$. Points represent behavioral data and shading represents model fits. (B) Proportion of trials in which the right item was selected, separated by whether the last fixation before the report was on the left (purple) or the right (green) item. (C) Mean response time as a function of the value difference between the two items. (D) Residual response time (after subtracting the contribution of Δr) as a function of the sum of the value of the two items presented in the trial $\left(\text{Σ}r\right)$. Error bars indicate standard error of the mean (s.e.m.) across trials. (E) Probability that the decision maker is looking at the item that was ultimately chosen, plotted relative to the time since the two items were presented on the screen (left) and the response time (right). Error bands indicate 95% confidence intervals for the mean across participants. In the stimulus-aligned plot, the data are shown from the first moment that one of the two items is fixated on in at least 50% of the trials, which is ∼0.25 s. (F) MELFB for data (black) and model (orange). Same conventions as in Fig. 2. (G) Gaze bias for consistent and inconsistent choices. Same conventions as in Fig. 3. (H) Proportion of trials in which the left item was selected as a function of the difference in dwell time between the left and right items. Data (black) and model (orange) were grouped in deciles of the dwell difference, separately for each participant, and then averaged across participants. (I) Proportion of trials in which the item looked at first was selected, as a function of duration of the first dwell. Data (black) and model (orange) were grouped in deciles of the first dwell duration, separately for each participant, and then averaged across participants. In all panels except panel B and G, data are shown in black and model simulations are shown in orange. Error bars and error bands, unless otherwise noted, show the standard error of the mean (s.e.m.) across participants (N=39 for both model and data).

We fit the *PDG* model to the choice and response time data from Krajbich et al. (2010). Fig. 5A and C show the proportion of trials in which participants chose the item on the right and the average RT as a function of Δr. The *PDG* model provides a good fit to the choice and RT data.

The model also accounts for MERT—the tendency to make faster decisions when the items being compared are overall more desirable, even when the value difference between them is the same (Smith and Krajbich, 2019; Sepulveda et al., 2020; Ratcliff et al., 2018). To illustrate this effect in Krajbich’s data, we fit a bell-shaped curve to the relation between RT and Δr, and computed residuals of RT by subtracting from each trial’s RT the expectation given by the best-fitting bell-shaped curve (see Fig. S1 for an illustration of the method). We then analyzed how the residuals of RT correlated with Σr. This correlation was negative, indicating that responses were faster when Σr was higher. That is, the data show a magnitude effect on RT (Fig. 5D). Note that the analysis of RT residuals rather than raw RT is necessary because of the positive correlation between Δr and Σr present in the data.

The *DDM* has been considered incapable of explaining the magnitude effect on RT because in the most common version of the *DDM* the drift rate depends only on the difference in value between options and ignores their absolute value, leading to the erroneous prediction that the response time for a given Δr would be independent of Σr. The *PDG* model captures the magnitude effect because the variance of the momentary evidence is allowed to change with Σr (Eq. 5) (Ratcliff et al., 2018). For the best-fitting model, the variance of the momentary evidence increased with Σr (Table S1). Because higher variance leads to faster responses (Zylberberg et al., 2016), the model displays a magnitude effect on RT, similar to the data (Fig. 5D).

In the *PDG* model, attention has no causal influence on choice. Yet, it successfully accounts for several features of the observed association between gaze and choice. We compared the probability of choosing the right item—as a function of Δr —on trials in which the last fixation was on the right versus the left item. The model predicts a systematic relation between the last fixation and choice that closely mirrors what is observed in the data (Fig. 5B).

In the *PDG* model, a gaze bias toward the chosen item can only occur during the non-decision time between choice commitment and report, giving rise to a *gaze cascade* effect—the observation that the probability of looking at the ultimately chosen item aligned to the response increases gradually over time (Fig. 5E). This gradual increase is explained as the average of step-like events (saccades to the chosen item) that occur at different times with respect to the response time. The variation in timing is due to trial-to-trial variability in non-decision time (captured by the model parameter $\sigma_{\text{nd}}$, see Methods) and in $\tau_{\text{e}}$. Since the model was fit to maximize the likelihood of its parameters based on the choice and RT data, without using gaze information, the gaze cascade effect can be considered a prediction of the model.

The *PDG* model also correctly predicts the two novel behavioral observations shown in Fig. 2 and Fig. 3. In the *PDG* model, there is no significant change in the strength of the last-fixation bias as a function of the overall value of the alternatives, consistent with the experimental data (Fig. 5F). This is because, in the model, the probability of directing gaze to the covertly chosen item is independent of variables affecting the decision process, like Δr or Σr.

The model also correctly predicts that ΔDwell does not depend on the consistency of the choice. In the model, ΔDwell is often positive because gaze is directed to the covertly chosen item and because $\tau_{\text{e}}$ is usually smaller than $\tau_{\text{m}}$. These factors do not depend on the consistency of the choice, resulting in similar values of ΔDwell for consistent and inconsistent choices (p = 0.27, Eq. 13, $H_{0}:\beta_{c}=0$)(Fig. 5G).

### The *gaze-cascade* effect continues after the choice report

In the *PDG* model, the bound crossing may have occurred hundreds of milliseconds before the choice is reported, and the two events are not time-locked due to variability in $\tau_{\text{m}}$ and $\tau_{\text{e}}$. The model posits that the gaze cascade arises because shifting gaze to the chosen item takes time, and the likelihood of having completed that shift increases with time elapsed since boundary crossing. By this logic, the model predicts an even further increase in the probability of looking at the chosen item immediately after the choice report.

**Figure 6.**
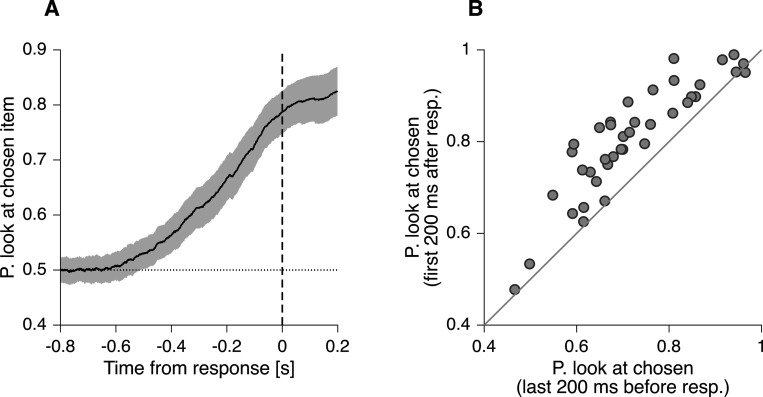
Gaze allocation after the choice report (A) Probability that the decision maker is looking at the item that was ultimately chosen, aligned to RT. This probability increases even after the choice report. Error bands indicate 95% confidence intervals for the mean across participants. (B) Proportion of time that the decision maker is looking at the item that was ultimately chosen, calculated for the last200 ms before the response (abscissa) and for the first 200 ms after the response (ordinate). Each data point represents one participant. Proportions were calculated as the sum of the time spent looking at the chosen item divided by the time spent looking at either one of the items (i.e., we exclude the times when the gaze was not directed at one of the two items).

To test this prediction, we examined the gaze allocation after the choice report. Fig. 6A shows the probability of looking at the chosen item, as a function of time, aligned to the response. The probability of looking at the chosen item continues to increase after the choice report. This can be seen more clearly in Fig. 6B, which shows the probability of looking at the chosen item during the 200 ms immediately before and after the choice report. For most participants, the probability is greater after the choice report (p *<* 10^−7^, Wilcoxon signed-rank test).

That is, a common process—directing the gaze to the chosen item—may explain the allocation of gaze both immediately before and after the choice report. In contrast, models that posit an exclusively intra-decision effect of attention on choice must then explain the allocation of gaze after the response as a separate process (e.g., directing gaze to the chosen item), or by assuming that individuals continue to evaluate the decision alternatives even after the choice report.

### Limitations of the *PDG* model

Despite the ability of the *PDG* model to explain many aspects of the data, other aspects are not as well captured. In the *PDG* model, choice accuracy is unaffected by fixation patterns prior to crossing the decision threshold. That is, whether decision-makers focus more on the higher-value item or distribute their attention evenly between the options, the model’s predictions for choice and RT remain unchanged. This invariance arises from the lack of a causal relation between gaze and the decision-making process. As a result, the *PDG* model makes no concrete predictions for the gaze pattern before choice commitment.

In simulated data—where dwell durations are independent of the value of the alternatives—the *PDG* model fails to explain certain experimental findings that demonstrate an association between dwell duration and choice (Krajbich et al., 2010; Callaway et al., 2021). We highlight this limitation with two analyses. The first examines the likelihood of choosing the item on the right as a function of the difference in dwell time between the right and left items. Both empirical data and *PDG* model simulations reveal a positive association, but this relation is steeper in the data (Fig. 5H). That is, ΔDwell is more predictive of choice in the experimental data than in the model, suggesting that decision-makers may fixate longer on the eventually chosen item even before covert choice commitment.

**Figure 7.**
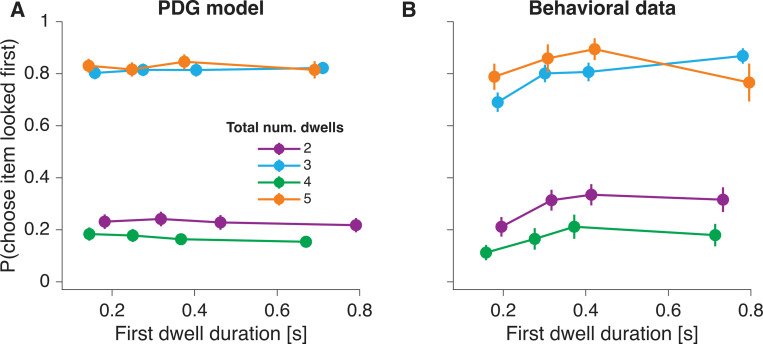
Association between first-dwell duration and choice probability (A) Model-predicted probability of choosing the option that was fixated first, as a function of the duration of the first dwell. Trials are grouped by the total number of dwells (2–5), shown in separate colors. Data were binned into quartiles of first-dwell duration and then averaged across participants. Error bars indicate s.e.m. (B) Same analysis as A, for the behavioral data.

The *PDG* model also fails to capture the relation between the duration of the first dwell and choice. Fig. 5I shows the proportion of trials in which decision-makers selected the initially fixated option as a function of the first dwell duration. This relation is steeper in the data than in the model. The *PDG* model does predict a positive relation between the duration of the first dwell and choice, but this is only because the bound is crossed during the first dwell on some trials. On trials with more than one dwell, the *PDG* model predicted no significant association between first-dwell duration and choice probability (all *𝑝 > .*05, likelihood-ratio tests; Eq. 14, Fig. 7A). In contrast, the empirical data revealed a significant positive association for shorter dwell-count conditions. Specifically, longer first-dwell durations significantly increased the probability of choosing the first-fixated item in 2-dwell (p = .013) and 3-dwell trials (p = .026), whereas this relationship was not significant for 4-dwell (p = .72) or 5-dwell trials (p = .96; Fig. 7B).

### Additive intra-decision attention also fails to explain the gaze–choice association

Alternative formulations of the *aDDM* posit that attention modulates value additively, rather than multiplicatively. We therefore asked whether a model with an additive, within-decision influence of attention can explain the novel empirical observations. In this additive intra-decisional attention model, the decision variable evolves according to

(6) $$x_{t}=x_{t-1}+d\left(r_{\text{left}}-r_{\text{right}}+s\omega \right)+\epsilon_{t},$$

where s=+1 when attention is directed to the left item and s=−1 when it is directed to the right item. In this formulation, the drift rate shifts by +ω or −ω depending on which item is attended, in contrast to the *aDDM*, where the unattended item’s value is discounted multiplicatively.

We fit both the multiplicative (*aDDM*) and additive model variants to the data from Krajbich et al. (2010), using identical fitting procedures. Model fitting was performed using the empirically observed sequence and duration of fixations on each trial (as in Li and Ma, 2021). To assess parameter identifiability, we simulated data using the best-fitting parameters and re-fit the models to these simulated datasets. The recovered parameters closely matched the generating values, regardless of whether attention acted additively or multiplicatively (Figs. S3 and S4). Across participants, both models provided comparable fits to the data: the additive and multiplicative models yielded similar BIC values (Fig. S5).

**Figure 8.**
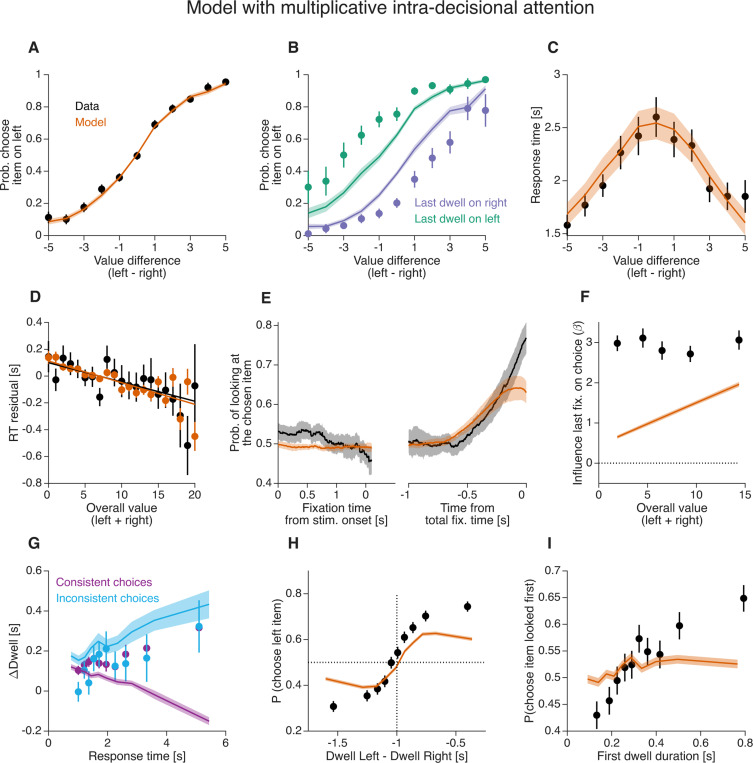
Human behavior and fits of the *aDDM* Same conventions as in Fig. 5. In panel E, only periods during which the participant is fixating one of the two items are included, as the drift rate in the *aDDM* is undefined otherwise. Accordingly, the plot shows time relative to fixation (i.e., elapsed fixation time), rather than absolute elapsed time as in Fig. 5.

**Figure 9.**
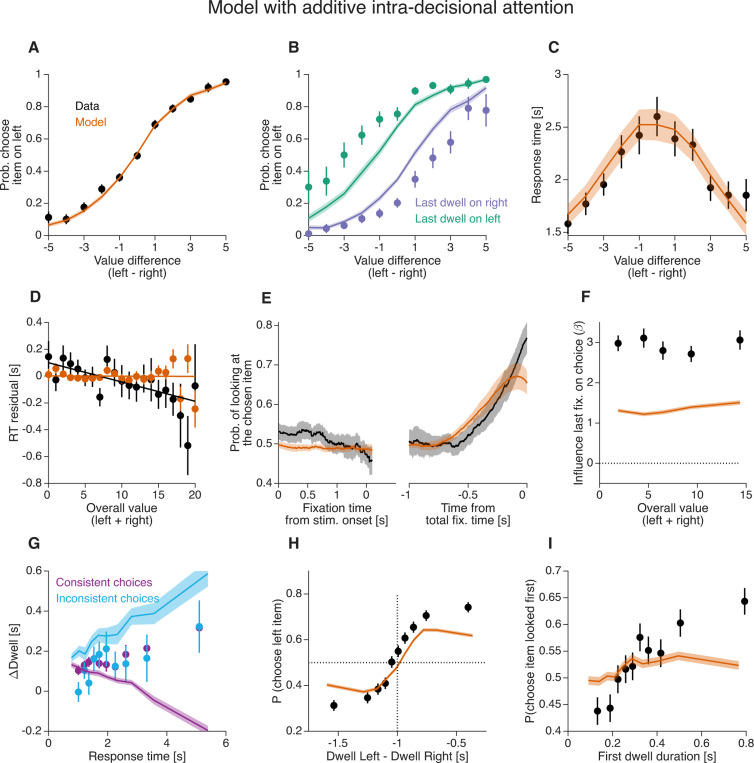
Human behavior and fits of a model with additive intra-decisional attention Same conventions as in Fig. 8.

Both the multiplicative and additive variants reproduced the main behavioral patterns, including the psychometric function (Fig. 8A; Fig. 9A), the chronometric function (Fig. 8C; Fig. 9C), and the gaze cascade effect (Fig. 8E; Fig. 9E). However, only the multiplicative model captured the observed dependence of response time on overall value (Fig. 8D; Fig. 9D), as has been shown by Smith and Krajbich (2019). Some aspects of the data were less accurately reproduced. In particular, both multiplicative and additive models underestimated the influence of the last fixation on choice (Fig. 8B; Fig. 9B). This discrepancy is larger than that reported by Krajbich et al. (2010). This is explained by the fact that their simulations assumed a uniform distribution of item-value pairs, resulting in a higher proportion of high-value trials than in the empirical dataset. Such a design amplifies value-dependent attentional effects, thereby increasing the apparent influence of the last fixation. As noted by Li and Ma (2021) and Callaway et al. (2021), assumptions about value distributions can substantially affect model behavior. Importantly, this limitation is not specific to our fitting approach: even when simulating the original *aDDM* using parameters from Krajbich et al. (2010), the model underestimates the effect of the last fixation when empirical value distributions are used (Fig. S7B). It is worth noting that Li and Ma (2021) analyzed the same dataset using a similar framework. In their report, the *aDDM* appears to reproduce the psychometric function conditioned on the last fixation more accurately, without the underestimation observed here (Fig. 8B). However, this difference arises from an analysis error in Li and Ma (2021), which led to misidentification of the last fixation in approximately 25% of trials (Wei Ji Ma, personal communication with Ariel Zylberberg). Overall, we conclude that neither the additive nor the multiplicative variants of the *aDDM* fully capture all empirical effects. Most notably, both fail to reproduce the observed association between choice consistency and ΔDwell.

### Combined intra- and post-decision attention

Neither a purely intra-decision nor a post-decision account of the gaze-choice association fully captures the behavioral data. We therefore considered the possibility that a combination of the two accounts provides a better explanation. Westbrook et al. (2020) proposed that the influence of attention on choice evolves within each trial: from an initial *multiplicative* influence, to a subsequent *additive* influence indicative of attention shifting toward the covertly chosen item.

We assessed whether a model incorporating both attentional mechanisms better explains the data, specifically regarding the effect of magnitude on the last fixation bias (MELFB) and the difference in ΔDwell between consistent and inconsistent choices. We combined elements from the *aDDM* and *PDG* models. Prior to committing to a choice, decision dynamics follow the *aDDM* framework—the value of the unattended option is multiplicatively discounted and evidence accumulation terminates upon reaching a threshold at ±B. Following threshold crossing, the gaze shifts to the covertly chosen item after a delay $\tau_{\text{e}}$, as described in the *PDG* model.

**Figure 10.**
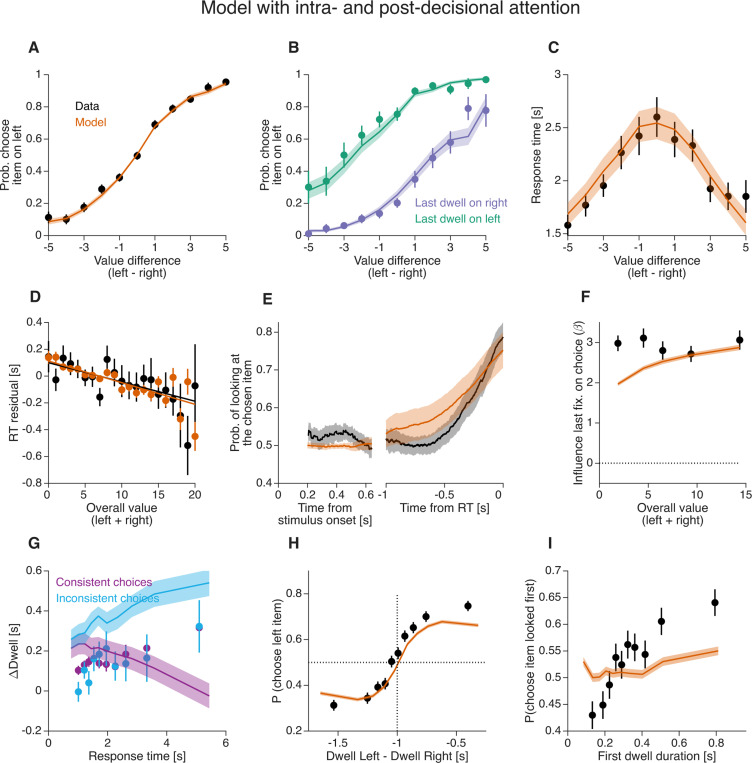
Human behavior and simulations of the combined *aDDM* and *PDG* model Starting from the *aDDM* simulations in Fig. 8, we added two features from the *PDG* model: (i) a gaze shift to the chosen item occurring with a delay $\tau_{\text{e}}$ after bound crossing, and (ii) a sensory delay $\tau_{\text{s}}$ between stimulus onset and the start of evidence accumulation. Same conventions as in Fig. 5.

Incorporating these post-decisional gaze shifts into the *aDDM* (Fig. 5) improves the model’s ability to explain the dependency between the last dwell, overall value, and choice (compare Fig. 10B,H and Fig. 8B,H). However, this hybrid approach still (*i*) inaccurately predicts that the gaze bias is stronger for inconsistent than for consistent choices (Fig. 10G), and (*ii*) displays a weaker association between the duration of the first fixation and choice, compared to what is observed in the data (Fig. 10I). From this we conclude that the combination of intra- and post-decision attentional mechanisms does not fully account for the behavioral data.

### Drift-rate variability across trials

None of the models that treat attention as an intra-decisional process were able to account for the difference in gaze bias between choices consistent versus inconsistent with stated preferences (e.g., Fig. 8G). We considered the possibility that inter-trial variability in the drift rate (e.g., Ratcliff and McKoon, 2008) could explain this discrepancy.

To test this, we fit a variant of the *aDDM* in which additive Gaussian noise corrupts the drift rate on each trial. The behavioral data and model fits are shown in Fig. S6. The model still incorrectly predicts a larger gaze bias for inconsistent choices than for consistent choices (Fig. S6G). The model also failed to capture the relation between first-dwell duration and choice (Fig. S6I).

**Figure 11.**
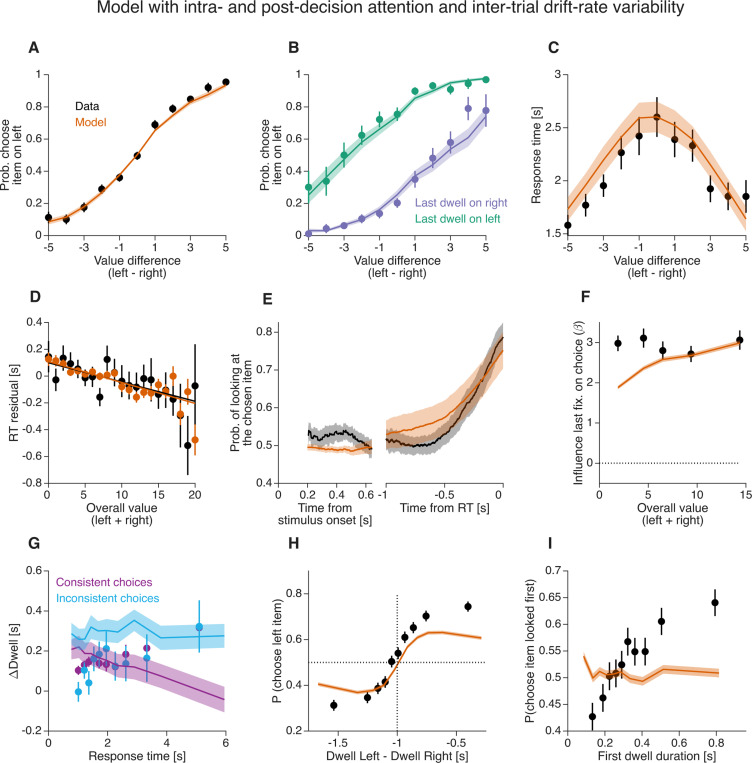
Model with intra- and post-decisional attentional effects, and inter-trial variability in the drift-rate Same as Fig. 10 but adding inter-trial variability in the drift-rate across trials. Same conventions as in Fig. 5.

To assess whether post-decisional processes might improve the model’s match to the data, we incorporated a post-decision gaze shift mechanism, similar to that in the *PDG* model. Even with this post-decisional mechanism the model predicts a larger gaze bias for inconsistent than for consistent choices, unlike what is observed in the data (Fig. 11G), and still fails to capture the observed association between first-dwell duration and choice (Fig. 11I).

We also explored an alternative implementation of inter-trial drift-rate variability, in which the item values— rather than the drift rate—are perturbed by additive Gaussian noise that remains constant within a trial. This approach is intended to capture the possibility that the values reported during the rating phase may differ from those used in the choice phase (c.f. Zylberberg et al., 2024). However, this variant did not provide a better fit to the behavioral data than the previous implementation (Fig. S8B,F-I).

For thoroughness, we also illustrate predictions from the models of Callaway et al. (2021) (Fig. S9) and Jang et al. (2021) (Fig. S10). Direct comparisons between these models should be interpreted cautiously due to variations in fitting methods: Callaway et al. (2021) fit their model simultaneously to two- and three-choice datasets, and Jang et al. (2021) manually selected parameters to approximate Krajbich et al. (2010)’s findings. Despite these methodological differences, it is evident that none of these models fully captures the comprehensive set of behaviors observed in this simple binary-choice task.

### Generalization to other food-choice datasets

**Figure 12.**
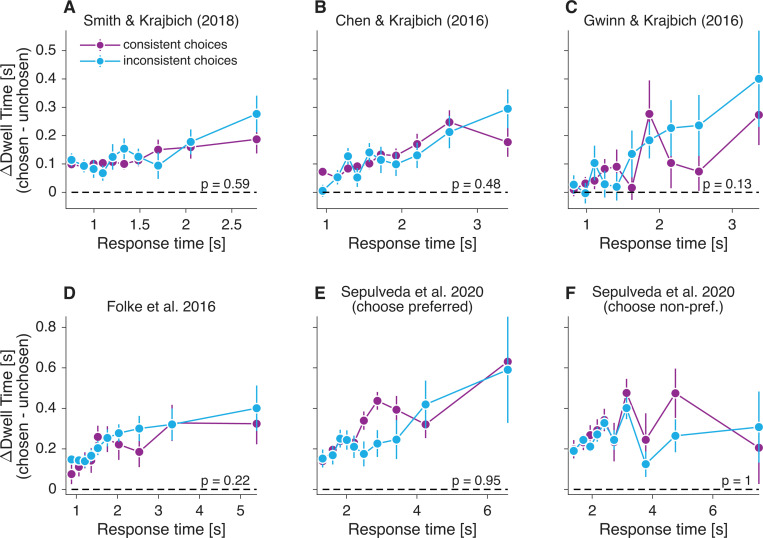
Difference in looking time for consistent and inconsistent choices in other datasets Same analysis as shown in Fig. 3, for six additional datasets. Each panel shows the difference in looking time between the chosen and the unchosen item (ΔDwell), as a function of response time, computed separately for choices that were consistent and inconsistent with stated valuation. The corresponding dataset is indicated in each panel. In Sepulveda et al. (2020), participants either selected the item they preferred or the one they did *not* prefer. We analyzed the two variants separately. Plotting conventions match those in Fig. 3.

We examined whether the absence of a difference in dwell time for consistent versus inconsistent choices (Fig. 3) generalizes to other food-choice datasets. We analyzed six publicly available datasets from tasks closely resembling that of Krajbich et al. (2010), reported in Smith and Krajbich (2018), Chen and Krajbich (2016), Gwinn (2016), Folke et al. (2016), and Sepulveda et al. (2020). Across all datasets, we found no significant difference in ΔDwell between choices that were consistent versus inconsistent with participants’ stated preferences (p > 0.12 for all datasets, one-tailed t-test; Eq. 13, Fig. 12), replicating the pattern observed in the data of Krajbich et al. (2010) (Fig. 3B).

## Discussion

We found post-decision signatures in the choice-RT-gaze data, suggesting that the association between gaze and choice, especially late in the trial, is not exclusively formative. Models positing an intra-decision (i.e., formative or constructive) multiplicative effect of attention on value—such as the attentional drift-diffusion model (*aDDM*)—predict that: (*i*) the last-fixation bias should be stronger when the items under consideration are overall more desirable, and (*ii*) ΔDwell—the difference in time spent looking at the chosen versus unchosen item—should be greater for inconsistent than for consistent choices. This is because inconsistent choices, in such models, benefit disproportionately from the attentional amplification of value. The data do not support these predictions (Fig. 2 and Fig. 3).

Instead, these observations are better explained by a *post-decision* account of the gaze-choice association—that is, one in which gaze shifts to the selected item *after* a covert commitment to a choice. The *PDG* model captures many empirical observations, including the last-fixation bias and the gaze cascade effect.

To account for the effect of overall value $\left(\text{Σ}r\right)$ on RT (Smith and Krajbich, 2019), the *PDG* model relies on the assumption that the variability of value representations increases with the values themselves (Ratcliff et al., 2018). This assumption is supported by neurobiological evidence. In the cortex, more desirable options tend to evoke higher firing rates (Platt and Glimcher, 1999; Padoa-Schioppa and Assad, 2006), and neural signal variance typically scales with its mean (Tolhurst et al., 1981). If the two value representations are independent, the variance of the momentary evidence, Δr, should therefore increase in proportion to Σr. In contrast, the *aDDM* explains the same relation between overall value and RT more directly—as a consequence of the multiplicative effect of attention on value—and thus does so more parsimoniously (Krajbich et al., 2010). Moreover, empirical results show that higher overall value leads to both faster and more accurate choices, arguing against the notion that sensitivity decreases with value. Instead, participants appear to invest additional effort in high-value decisions, which may offset any increase in variability (Shevlin et al., 2022).

Further, the *PDG* model underestimates the observed association between both early dwell-time and choice probability, as well as the association between ΔDwell and choice probability. Inspired by Westbrook et al. (2020), we therefore examined a *hybrid model* in which attention initially exerts a causal influence on the choice process and subsequently reflects the chosen option. This mixed model reintroduced some of the same limitations seen in the *aDDM*—most notably, its inability to account for the null effect of overall value on the last-fixation bias (MELFB) and the similarity in gaze bias across consistent and inconsistent decisions. We conclude that neither of these models is fully able to account for the data from the food-choice task.

Similar shortcomings to those observed in the *aDDM* were observed in models that derive attention-choice associations from optimal or near-optimal policies. Specifically, Callaway et al. (2021) and Jang et al. (2021) formalized the decision process as a partially observable Markov decision process (POMDP), in which attention enhances either the quantity (Callaway et al., 2021) or quality (Jang et al., 2021) of evidence about item value. To explain the gaze-choice association, both models assume that priors over item values are miscalibrated, such that decision-makers underestimate the true values. As a result, less-attended items are more biased toward zero, since less-attended items are more influenced by the miscalibrated prior. However, simulations based on these models fail to reproduce key empirical findings, including the association between ΔDwell, decision consistency, and RT (Figs. 2, 3, S9 and S10).

The possibility that the gaze-choice association is partially post-decisional may help reconcile discrepancies between studies using free-viewing paradigms and those employing causal manipulations of attention. Common approaches to manipulating attention include limiting exposure duration (Frömer et al., 2022), interrupting trials based on gaze duration (Pärnamets et al., 2015; Newell and Le Pelley, 2018; Tavares et al., 2017; Pleskac et al., 2023), and cueing spatial attention (Störmer and Alvarez, 2016; Gwinn et al., 2019). A meta-analysis of the effects of visual attention on binary consumer choice (Bhatnagar and Orquin, 2022) found that these manipulations typically shift choice probabilities by ∼2–4% from a 50% baseline (cf., Tavares et al., 2017). These small effects contrast sharply with the large effects estimated by fits of the *aDDM*, which often posit a 70% discount of unattended items (Krajbich et al., 2010). The discrepancy between model predictions and the results of the attention-manipulation studies may arise from attempting to account for post-decision gaze-choice correlations using models that assume that the gaze-choice association is exclusively intra-decisional.

A post-decision gaze-choice association may offer a parsimonious explanation for differences in gaze behavior between tasks in which items are either chosen or rejected. In “choose” tasks, participants select the preferred item; in “reject” tasks, they exclude the less preferred item. Although logically equivalent in binary choice, the gaze is directed more to the preferred item in “choose” tasks and to the non-preferred item in “reject” tasks (Sepulveda et al., 2020; van der Laan et al., 2015; Mitsuda and Glaholt, 2014; Nittono and Wada, 2009). This difference has been explained by a variant of the *aDDM* in which attention modulates the integration of goal-relevant evidence (Sepulveda et al., 2020). Without ruling out this possibility, our results suggest that the difference in gaze allocation between “choose” and “reject” tasks arises because toward the end of the trial the gaze is directed to the selected option, regardless of whether it is to be accepted or rejected.

In this work, we focused on a simple yet widely used class of models in which decisions are based on comparing noisy value signals assigned to each item, and where explicit ratings are assumed to reflect the true underlying value of those items. An alternative class of models proposes that decisions arise from comparisons along individual feature dimensions, rather than at the level of the item as a whole (Tversky, 1972; Roe et al., 2001; Usher and McClelland, 2004; Busemeyer and Townsend, 1993; Summerfield and Tsetsos, 2015; Shadlen and Shohamy, 2016; Lichtenstein and Slovic, 2006; Johnson et al., 2007; Lee and Hare, 2023; Yang and Krajbich, 2023; Fisher, 2021). In food choice tasks, for example, relevant features might include expected satiety, caloric content, tastiness, saltiness, and so on (Rangel and Hare, 2010; Sullivan et al., 2015; Rramani et al., 2020; Suzuki et al., 2017). Attention is thought to fluctuate across these features, updating a decision variable for each alternative as different dimensions are sampled. Which dimensions are evaluated—and the weight assigned to each—may depend on the items being compared, past experience, or the broader decision context (Noguchi and Stewart, 2018; Roe et al., 2001; Juechems and Summerfield, 2019; Zylberberg et al., 2024; Lee and Pezzulo, 2022; Trueblood et al., 2014; Bhatia, 2013). As a result, the desirability of an item during the decision process may differ from that reported during the rating phase. A choice may appear inconsistent relative to initial ratings, but not relative to the specific features that were actually attended during deliberation. Similarly, if the ratings themselves are noisy and do not perfectly reflect subjective value, then some choices labeled as inconsistent may, in fact, be consistent with the decision-maker’s true preferences. Incorporating noise into the rating process would blur the distinction between consistent and inconsistent choices, potentially improving the alignment between model and data. These speculative ideas remain to be tested in future work.

Overall, our findings suggest that the association between attention and choice is not fully captured by models in which attention plays a purely causal role during decision formation. The data suggest an additional post-decision association, in which attention reflects, rather than shapes, the covert choice. While this does not preclude a formative influence of attention at earlier stages, it highlights the importance of considering the temporal dynamics of commitment when interpreting gaze patterns.

## Methods

### Food-choice task

We reanalyzed the data from the experiment reported in Krajbich et al. (2010). The task was completed by 39 participants. The experiment consisted of two phases. In the first (‘rating’) phase, participants were asked to indicate how much they would like to consume each item after the experiment. This was done using an on-screen slider bar (scale −10 to 10). 70 items were rated in this phase. In the next (‘choice’) phase, participants were presented with pairs of previously rated items and had to choose which one they would prefer to eat at the end of the experiment. Items that received a negative rating were excluded from the choice phase. Each item could be repeated up to six times. Choice trials were created such that the difference in value between items was less than or equal to five (except for five participants for whom this condition could not be met, see Krajbich et al. 2010). Choices were made by pressing the left or right arrow keys on the keyboard. Prior to the presentation of the pair of items, participants had to maintain fixation on a central spot for two seconds. After participants indicated their choice, a box was drawn around the chosen item, which remained visible for one second. Each participant completed 100 trials. Details of the experiment can be found in the original publication.

### *PDG* model

In the *PDG* model, choice and decision time are determined by the state of a scalar decision variable, x. The decision variable is the cumulative sum of samples from a normal distribution with mean μdt and variance $\sigma^{2}dt$, and thus its evolution is described by the stochastic differential equation,

(7) dx=μdt+σdW,

where W is the standard Wiener process and x(t=0)=0. The drift rate μ is given by

(8) $$\mu =\kappa \left(r_{\text{right}}-r_{\text{left}}\right),$$

where $r_{\text{left}}$ and $r_{\text{right}}$ are the ratings assigned to the items presented on the left and right of the screen. The variance of the momentary evidence, $\sigma^{2}$, scales linearly with the sum of the ratings (Eq. 5).

The accumulation process ends when the decision variable, x(t), reaches one of two bounds positioned symmetrically around zero, at ±B. The left (right) item is chosen when the decision variable reaches the upper (lower) bound. This first passage time establishes the decision time. The RT is the sum of the decision time plus the non-decision latencies, $\tau_{\text{nd}}$. We assume that $\tau_{\text{nd}}$ is Normally distributed with mean $\mu_{\text{nd}}$ and standard deviation $\sigma_{\text{nd}}$.

The accumulation process starts after a short sensory delay, $\tau_{\text{s}}$ (Fig. 4), which is part of the non-decision latency. We used a fixed value of $\tau_{\text{s}}=0.3$ s for all participants. The remaining non-decision time $\left(\tau_{\text{m}}=\tau_{\text{nd}}-\tau_{\text{s}}\right)$ is assigned to the time between crossing a decision bound and reporting the choice.

After crossing a bound, gaze is directed to the selected item. The time taken to switch gaze to the chosen item has an associated non-decision latency of $\tau_{\text{e}}$. This latency is assumed to follow a truncated Normal distribution with a mean of $\mu_{e}=0.35$ seconds and a standard deviation $\sigma_{e}$ of one-third of the mean, truncated to ensure non-negative values. Since $\tau_{\text{e}}$ is usually smaller than $\tau_{\text{m}}$, the gaze is informative about the choice.

We fit the model parameters $\lambda =\left\{\kappa ,B,\gamma ,\mu_{\text{nd}},\sigma_{\text{nd}}\right\}$ using maximum likelihood to the choice and RT data from each trial. Fits were performed independently for each participant. The log-likelihood of the parameters is given by:

(9) $$\log L_{\lambda }=\sum_{i=1}^{n}\log p\left({\text{choice}}^{\left(i\right)},{\text{RT}}^{\left(i\right)}|\Delta v^{\left(i\right)},\Sigma v^{\left(i\right)},\lambda \right),$$

where the summation is over trials. The joint probability of choice and decision time was obtained by numerically solving the Fokker-Planck equation associated with the drift-diffusion process using the Chang-Cooper fully implicit method (Chang and Cooper, 1970; Zylberberg et al., 2016; Kiani and Shadlen, 2009). To obtain the joint probability distribution over choice and RT, we convolve the probability distribution of decision times with the distribution of non-decision times, given by a truncated normal distribution with parameters $\mu_{\text{nd}}$ and $\sigma_{\text{nd}}$ (the truncation constrains the non-decision times to be positive).

### Attentional drift-diffusion model

We simulated the *aDDM* (Eqs. 1 and 2) using the best-fitting parameters reported by Krajbich et al. (2010): $d=0.0002{\text{ms}}^{-1}$, σ=0.02, θ=0.3 and bounds B=±1. The response time was calculated as the sum of the decision time from the drift-diffusion process and a fixed non-decision time of $t_{\text{nd}}=0.355$ s, as in Smith and Krajbich (2019).

In the *aDDM* (as well as in the *DDM*), it is necessary to set one of the parameters d, σ, or B to a fixed value for the model parameters to be identifiable. In the original description of the *aDDM* (Krajbich et al., 2010), the upper and lower bounds were set to a fixed value of ±1. For consistency with the *PDG* model, we reformulated the *aDDM* so that the variance of the noise accumulated during one second of unbounded accumulation is equal to 1. Then the decision variable of the *aDDM*, x(t), evolves as in Eq. 7 with σ=1. Time is discretized in steps of dt = 0.001 s. The drift rate *𝜇* is equal to $\kappa \left(r_{\text{right}}-\theta r_{\text{left}}\right)$ when looking at the item on the right, and to $\kappa \left(\theta r_{\text{right}}-r_{\text{left}}\right)$ when looking at the item on the left, where κ is the signal-to-noise. A model equivalent to the original *aDDM* is obtained with the following parameters:

(10) $$\kappa =\frac{d}{\sigma }\sqrt{1000}=0.3162\;B=\pm \frac{1}{\sigma }\frac{1}{\sqrt{1000}}=\pm 1.5811\;\theta =0.3.$$

Simulating the *aDDM* requires modeling how attention alternates between the two items. We fit the empirically-observed dwell durations with log-normal distributions. Separate fits were conducted for the first and middle dwells. Middle dwells are those that were neither the first nor the last of the stimulus-viewing epoch. The fits provide a good match to the experimental data (Fig. S2). Each simulated trial of the *aDDM* begins by sampling from the first dwell distribution, followed by sampling from the middle dwell distribution until a decision threshold is reached. Similar to the experimental data, the first fixation has a 0.74 probability of being directed to the left item, and attention alternates between the two items thereafter. We simulated the same trials (same value pairs) as those completed by the participants, repeating each trial 10 times.

We fit three variants of the *aDDM* to individual participant data. In the first variant, attention has an additive effect on value, rather than a multiplicative effect (Fig. 9; see equations for the drift rate in the main text). In the second variant (Fig. S6), the drift rate exhibits inter-trial variability. Specifically, on each trial, the drift rate μ is given by $\kappa \left(r_{\text{left}}-\theta r_{\text{right}}\right)+v$ when fixating the left item, and by $\kappa \left(\theta r_{\text{left}}-r_{\text{right}}\right)+v$ when fixating the right item. Here, v is trial-specific noise drawn from a normal distribution with mean 0 and standard deviation $\sigma_{d}$, which is estimated from the data. Importantly, v is constant within a trial but varies across trials.

The third *aDDM* variant can be interpreted as alternative implementation of the one with inter-trial drift-rate variability. The noise does not directly affect the drift rate, but the items’ value. Specifically, the drift rate μ is given by $\kappa \left(\left(r_{\text{left}}+v_{1}\right)-\theta \left(r_{\text{right}}+v_{2}\right)\right)$ when fixating the left item, and by $\kappa \left(\theta \left(r_{\text{left}}+v_{1}\right)-\left(r_{\text{right}}+v_{2}\right)\right)$ when fixating the right item. Here, $v_{x}$ represents trial-specific noise, drawn from a normal distribution with mean 0 and standard deviation $\sigma_{d}$. This formulation reflects the idea that the value reported in the rating phase may deviate from the item’s “true” underlying value (c.f., Zylberberg et al. (2024); Polania et al. (2019)).

Additionally, we simulated the *aDDM* using the best-fitting parameters reported in previous studies by Krajbich et al. (2010) and Smith and Krajbich (2019) (Fig. S7).

The core parameters of the *aDDM* are λ={κ,B,θ}, corresponding respectively to the signal-to-noise ratio, the decision bound height, and the value-scaling factor applied to the unattended option. In the model with inter-trial drift-rate variability, $\sigma_{d}$ is added to capture the standard deviation of the drift-rate noise. The models are fit to maximize the likelihood of the parameters given the choice and decision time (DT), and given the sequence and duration of the dwells observed on each trial:

(11) $$\log L_{\lambda }=\sum_{i=1}^{n}\log p\left({\text{choice}}^{\left(i\right)},{\text{DT}}^{\left(i\right)}|v_{\text{left}}^{\left(i\right)},v_{\text{right}}^{\left(i\right)},{\text{D}}^{\left(i\right)},\lambda \right),$$

where ${\text{D}}^{\left(i\right)}$ is the sequence and duration of dwells observed on trial i, and ${\text{DT}}^{\left(i\right)}$ is the decision time on trial *𝑖* that is assumed to be equal to the sum of the dwells on either of the two items. The joint probability of choice and decision time was computed via numerical approximations of the corresponding Fokker-Planck (FP) equation. Unlike the special case where θ=1, here the drift rate varies with the focus of attention. As a result, solving the FP equation becomes more computationally demanding, since the probability density of the decision variable must be computed separately for each trial, given the trial-specific stochastic fluctuations in gaze. Numerical solutions were obtained using a fully implicit method (Chang and Cooper, 1970), propagating the probability density of the decision variable over time adjusting the drift rate depending on the focus of gaze.

For the model with inter-trial variability in the drift-rate, we discretized the distribution of drift perturbations into a finite number of bins. Specifically, we drew $n_{\text{bins}}=11$ quantile-based samples from a zero-mean Gaussian distribution with standard deviation $\sigma_{\text{drift}}$, using the midpoint of each quantile as a representative value. We numerically solved the Fokker–Planck equation independently for each of these bins. The final probability distribution over choice and decision times was computed as the average (uniform-weighted) across the solutions for each bin, effectively marginalizing over the distribution of drift perturbations.

We use the best-fitting parameters to simulate the *aDDM* independently for each participant. From the simulations we obtain a choice and decision time for each trial. Response times are estimated as the decision time plus mean non decision time, $\mu_{nd}$, defined as the trial-average RT of each participant minus the trial-average decision time obtained from the model simulations.

### Model simulations

Simulations of the *PDG* model (Fig. 5) and *aDDM* (Figs. 8 to 10) were made using the same trials (same value pairs) that participants completed, with each trial repeated 10 times. Dwell durations were randomly sampled from log-Normal distributions fit to the duration of the dwells (Fig. S2). First and subsequent dwells were fit separately. The probability of sampling the left item first was set to 0.74 to match the value obtained from the experimental data.

### Combined *aDDM*-*PDG* model

The combined *aDDM*-*PDG* model (Fig. 10) builds on the *aDDM* fit to single-participant data. We add to the data simulated with the *aDDM* (Fig. 8) two aspects of the *PDG* model: (i) a sensory delay between the onset of the food items and the start of the evidence accumulation process of $\tau_{\text{s}}$, and (ii) another delay $\tau_{\text{e}}$ between the time at which a bound is crossed and the time at which the gaze is directed to the chosen item (truncated such that $\tau_{\text{e}}$ is non-negative). Parameters $\tau_{\text{s}}$, $\mu_{e}$ and $\sigma_{e}$ were set to 0.25s, 0.2s and 0.05s respectively. The same approach and parameter values were used in the *aDDM* model with inter-trial variability in the drift rate (Fig. 11).

### Model fitting

Parameter optimization was performed using the Bayesian Adaptive Direct Search (BADS) method (Acerbi and Ma, 2017). Table S1, Table S2, Table S3 and Table S4 show the best-fitting parameters for the *PDG* model, the *aDDM*, the model with intra-decisional additive attention, and the *aDDM* with inter-trial drift-rate variability, respectively.

### Optimal decision models

The optimal model of Jang et al. (2021) has 5 free parameters: the cost of switching attention between items $\left(c_{s}\right)$, the cost, per second, of accumulating evidence $\left(c\right)$, the variance of the evidence sampling distribution $\left(\sigma_{X}^{2}\right)$, the variance of the prior distribution $\left(\sigma_{z}^{2}\right)$, and the relative information gain for attended vs. unattended items (κ). See Jang et al. (2021) for a detailed explanation of the model. We simulated 1280 trials per participant with the parameters that Jang et al. (2021) reported best replicated the human behavioral data: $c_{s}=0.0065$, c=0.23, $\sigma_{x}^{2}=27$, $\sigma_{z}^{2}=18$, κ=0.004. As in Jang et al., the prior mean over the items’ values, $\bar{z}$, was set to zero, which is lower than the true mean value of the items. The model requires this feature to produce a gaze bias (Jang et al., 2021).

We also analyzed the simulations of the optimal model developed by Callaway et al. (2021). The model has five free parameters: the standard deviation of the evidence sampling distribution, the cost of obtaining a sample, the cost of switching attention between items, the degree to which the prior over the item values is biased toward zero, and a ‘temperature’ parameter of a Boltzmann distribution, which controls the degree of stochasticity in the selection of the optimal policy. The simulations (N = 4,550,400 trials) with the parameters that best fit the human behavioral data were kindly provided by Frederick Callaway and are available at https://github.com/fredcallaway/optimal-fixations-simple-choice. A detailed explanation of the model and the fitting procedure can be found in the original publication (Callaway et al., 2021).

### Data analysis

The dashed lines in Fig. 2(top) were derived from the following logistic regression model:

(12) $$\mathrm{logit}\left[p_{\text{left}}\right]=\sum_{s}\left(\beta_{\text{Δ}r,s}\text{Δ}r+\beta_{0,s}\right)I_{s}+\beta_{\text{LastFix}}I_{\text{LastFix}}+\beta_{\text{LastFix},\Sigma r}I_{\text{LastFix}}\Sigma r$$

$I_{\text{LastFix}}$ takes a value of 1 if the left item was fixated on last and 0 if the right item was fixated last. The rightmost term captures the interaction of $I_{\text{LastFix}}$ with Σr ; the associated β is the slope of the dashed line in Fig. 2(top).

We fit the following linear regression model to test for an association between choice consistency $\left(c\right)$ and the difference in looking time between the chosen and unchosen items (ΔDwell):

(13) $$\text{Δ Dwell}=\sum_{i=1}^{N}\beta_{i}I_{i}+\sum_{i=1}^{N}\beta_{N+i}I_{i}RT+\beta_{c}c,$$

where N is the number of participants, $I_{i}$ is an indicator variable that takes the value 1 if the trial was completed by subject i and 0 otherwise, and c is equal to 1 for trials in which the higher-rated item was chosen, defined as a *consistent* choice, and and 0 for trials in which the lower-rated item was chosen, defined as an *inconsistent* choice. We used a one-tailed *t*-test to test whether $\beta_{c}$ is negative, that is, whether ΔDwell is larger for inconsistent than for consistent choices. When the use of a one-tailed test is not explicitly mentioned, we used two-tailed *t*-tests to test whether $\beta_{c}$ differed significantly from zero.

To test the hypothesis that there was no difference in the probability of looking at the chosen item before and after the choice report (Fig. 6), we used a Wilcoxon signed-rank test. We define two time epochs, one from −200 ms to 0 ms, and the other from 0 ms to 200 ms, relative to the time of the choice report. For each time epoch, we calculated the time each participant spent looking at the chosen item and divided it by the time spent looking at either item. We obtain two proportions per participant (Fig. 6B), which we subjected to a two-tailed Wilcoxon signed-rank test.

For the plots showing the probability of looking at the chosen item aligned to stimulus onset (e.g., Fig. 5E, left), we eliminated the gaze information from the 500 ms prior to the choice report. This step was taken to eliminate, from the stimulus-aligned plots, gaze effects that might be related to the response.

For the plots showing the psychometric function split by whether the last fixated item was the one on the left or right (e.g., Fig. 5B), we classified trials as ‘left item fixated on last’ and ‘right item fixated on last’ depending on which item was being looked at at the time of the choice report. We excluded trials in which the participant either was not fixating on one of the two relevant items at the time of the choice report or the direction of gaze could not be resolved (e.g., eye blinks). We repeated the analyses reclassifying trials according to which item was last looked at before the choice report regardless of when it occurred during the trial, and obtained nearly identical results.

To test the association between first-dwell duration and the probability of choosing the option that was fixated first (Fig. 7), we fit the following logistic regression model, separately for each total–dwell–count condition (2–5 total dwells):

(14) $$\text{logit}\left[p_{\text{match}}\right]=\beta_{0}+\beta_{1}D+\beta_{2}r_{\text{first}}+\beta_{3}r_{\text{other}}$$

where $p_{\text{match}}$ is the probability of choosing the item fixated first, D is the standardized first-dwell duration, and $r_{\text{first}}$ and $r_{\text{other}}$ represent the value ratings of the first-fixated and alternative items, respectively. Statistical significance of the first-dwell-duration coefficient was assessed using a likelihood-ratio test, comparing the full model to a reduced model that excluded the $\beta_{1}$ term.

To illustrate the gaze cascade effect (e.g., Fig. 5E), we filled a matrix of dimensions NumberOfTrials × NumberOfTimeSteps, with a 1 when participants were looking at the right item, a 0 when they were looking at the left item, and a NaN otherwise. The time step was 1 millisecond. We averaged this matrix across trials, ignoring the NaNs, first within participants and then across participants. For the RT aligned plots, we followed the same procedure after aligning each trial to the response time. For the *aDDM* and and Callaway’s optimal model, we followed a slightly different procedure, because in these models the drift-rate is undefined when participants are not looking at either of the two items. Therefore, we removed from each trial the times when the participant’s gaze was not directed to either snack item, and aligned the responses to the total fixation time rather than to the response time.

## Supplementary Material

Supplement 1

## References

[R1] Acerbi L, Ma WJ. Practical Bayesian optimization for model fitting with Bayesian adaptive direct search. arXiv preprint arXiv:170504405. 2017; .

[R2] Armel KC, Beaumel A, Rangel A. Biasing simple choices by manipulating relative visual attention. Judgment and Decision making. 2008; 3(5):396–403.

[R3] Bhatia S. Associations and the accumulation of preference. Psychological review. 2013; 120(3):522. 23607600 10.1037/a0032457

[R4] Bhatnagar R, Orquin JL. A meta-analysis on the effect of visual attention on choice. Journal of Experimental Psychology: General. 2022; . 10.1037/xge0001204 35849390

[R5] Busemeyer JR, Townsend JT. Decision field theory: a dynamic-cognitive approach to decision making in an uncertain environment. Psychological review. 1993; 100(3):432. 8356185 10.1037/0033-295x.100.3.432

[R6] Callaway F, Rangel A, Griffiths TL. Fixation patterns in simple choice reflect optimal information sampling. PLoS computational biology. 2021; 17(3):e1008863. 10.1371/journal.pcbi.1008863 PMC8026028 33770069

[R7] Cavanagh JF, Wiecki TV, Kochar A, Frank MJ. Eye tracking and pupillometry are indicators of dissociable latent decision processes. Journal of Experimental Psychology: General. 2014; 143(4):1476. 24548281 10.1037/a0035813 PMC4114997

[R8] Chang J, Cooper G. A practical difference scheme for Fokker-Planck equations. Journal of Computational Physics. 1970; 6(1):1–16.

[R9] Chen WJ, Krajbich I. Pupil dilation and attention in value-based choice. Unpublished manuscript, The Ohio State University. 2016; .

[R10] Fisher G. A multiattribute attentional drift diffusion model. Organizational Behavior and Human Decision Processes. 2021; 165:167–182.

[R11] Folke T, Jacobsen C, Fleming SM, De Martino B. Explicit representation of confidence informs future value-based decisions. Nature Human Behaviour. 2016; 1(1):0002.

[R12] Frömer R, Callaway F, Griffiths T, Shenhav A. Considering what we know and what we don’t know: Expectations and confidence guide value integration in value-based decision-making. PsyArXiv. 2022; . 10.1162/opmi.a.3 PMC12240722 40642140

[R13] Glaholt MG, Reingold EM. The time course of gaze bias in visual decision tasks. Visual Cognition. 2009; 17(8):1228–1243.

[R14] Gwinn R, Leber AB, Krajbich I. The spillover effects of attentional learning on value-based choice. Cognition. 2019; 182:294–306. 30391643 10.1016/j.cognition.2018.10.012

[R15] Gwinn RE, Attitudes and attention: How attitude accessibility and certainty influence attention and subjective choice; 2016.

[R16] Jang AI, Sharma R, Drugowitsch J. Optimal policy for attention-modulated decisions explains human fixation behavior. Elife. 2021; 10:e63436. 10.7554/eLife.63436 PMC8064754 33769284

[R17] Johnson EJ, Häubl G, Keinan A. Aspects of endowment: a query theory of value construction. Journal of experimental psychology: Learning, memory, and cognition. 2007; 33(3):461. 17470000 10.1037/0278-7393.33.3.461

[R18] Juechems K, Summerfield C. Where does value come from? Trends in cognitive sciences. 2019; 23(10):836–850. 31494042 10.1016/j.tics.2019.07.012

[R19] Kiani R, Shadlen MN. Representation of confidence associated with a decision by neurons in the parietal cortex. science. 2009; 324(5928):759–764. 19423820 10.1126/science.1169405 PMC2738936

[R20] Krajbich I. Accounting for attention in sequential sampling models of decision making. Current opinion in psychology. 2019; 29:6–11. 30368108 10.1016/j.copsyc.2018.10.008

[R21] Krajbich I, Armel C, Rangel A. Visual fixations and the computation and comparison of value in simple choice. Nature neuroscience. 2010; 13(10):1292–1298. 20835253 10.1038/nn.2635

[R22] Krajbich I, Lu D, Camerer C, Rangel A. The attentional drift-diffusion model extends to simple purchasing decisions. Frontiers in psychology. 2012; 3:193. 22707945 10.3389/fpsyg.2012.00193 PMC3374478

[R23] Krajbich I, Rangel A. Multialternative drift-diffusion model predicts the relationship between visual fixations and choice in value-based decisions. Proceedings of the National Academy of Sciences. 2011; 108(33):13852–13857. 10.1073/pnas.1101328108 PMC3158210 21808009

[R24] van der Laan LN, Hooge IT, De Ridder DT, Viergever MA, Smeets PA. Do you like what you see? The role of first fixation and total fixation duration in consumer choice. Food Quality and Preference. 2015; 39:46–55.

[R25] Lee DG, Hare TA. Evidence accumulates for individual attributes during value-based decisions. Decision. 2023; 10(4):330.

[R26] Lee DG, Pezzulo G. Choice-induced preference change under a sequential sampling model framework. bioRxiv. 2022; p. 2022–07. 10.1038/s41598-026-44610-5 PMC13149806 41865112

[R27] Li ZW, Ma WJ. An uncertainty-based model of the effects of fixation on choice. PLoS computational biology. 2021; 17(8):e1009190. 10.1371/journal.pcbi.1009190 PMC8389845 34398884

[R28] Lichtenstein S, Slovic P. The construction of preference. Cambridge University Press; 2006.

[R29] Link SW. The relative judgment theory of two choice response time. Journal of Mathematical Psychology. 1975; 12(1):114–135.

[R30] Mitsuda T, Glaholt MG. Gaze bias during visual preference judgements: Effects of stimulus category and decision instructions. Visual Cognition. 2014; 22(1):11–29.

[R31] Newell BR, Le Pelley ME. Perceptual but not complex moral judgments can be biased by exploiting the dynamics of eye-gaze. Journal of Experimental Psychology: General. 2018; 147(3):409. 29355370 10.1037/xge0000386

[R32] Nittono H, Wada Y. Gaze shifts do not affect preference judgments of graphic patterns. Perceptual and motor skills. 2009; 109(1):79–94. 19831089 10.2466/PMS.109.1.79-94

[R33] Noguchi T, Stewart N. Multialternative decision by sampling: A model of decision making constrained by process data. Psychological review. 2018; 125(4):512. 29952622 10.1037/rev0000102 PMC6022729

[R34] Padoa-Schioppa C, Assad JA. Neurons in the orbitofrontal cortex encode economic value. Nature. 2006; 441(7090):223–226. 16633341 10.1038/nature04676 PMC2630027

[R35] Pärnamets P, Johansson P, Hall L, Balkenius C, Spivey MJ, Richardson DC. Biasing moral decisions by exploiting the dynamics of eye gaze. Proceedings of the National Academy of Sciences. 2015; 112(13):4170–4175. 10.1073/pnas.1415250112 PMC4386374 25775604

[R36] Platt ML, Glimcher PW. Neural correlates of decision variables in parietal cortex. Nature. 1999; 400(6741):233–238. 10421364 10.1038/22268

[R37] Pleskac TJ, Yu S, Grunevski S, Liu T. Attention biases preferential choice by enhancing an option’s value. Journal of Experimental Psychology: General. 2023; 152(4):993. 36301270 10.1037/xge0001307

[R38] Polania R, Woodford M, Ruff CC. Efficient coding of subjective value. Nature neuroscience. 2019; 22(1):134–142. 30559477 10.1038/s41593-018-0292-0 PMC6314450

[R39] Rangel A, Hare T. Neural computations associated with goal-directed choice. Current opinion in neurobiology. 2010; 20(2):262–270. 20338744 10.1016/j.conb.2010.03.001

[R40] Ratcliff R. A theory of memory retrieval. Psychological review. 1978; 85(2):59.

[R41] Ratcliff R, McKoon G. The diffusion decision model: theory and data for two-choice decision tasks. Neural computation. 2008; 20(4):873–922. 18085991 10.1162/neco.2008.12-06-420 PMC2474742

[R42] Ratcliff R, Voskuilen C, Teodorescu A. Modeling 2-alternative forced-choice tasks: Accounting for both magnitude and difference effects. Cognitive psychology. 2018; 103:1–22. 29501775 10.1016/j.cogpsych.2018.02.002 PMC5911219

[R43] Roe RM, Busemeyer JR, Townsend JT. Multialternative decision field theory: A dynamic connectionst model of decision making. Psychological review. 2001; 108(2):370. 11381834 10.1037/0033-295x.108.2.370

[R44] Roitman JD, Shadlen MN. Response of neurons in the lateral intraparietal area during a combined visual discrimination reaction time task. Journal of neuroscience. 2002; 22(21):9475–9489. 12417672 10.1523/JNEUROSCI.22-21-09475.2002 PMC6758024

[R45] Rramani Q, Krajbich I, Enax L, Brustkern L, Weber B. Salient nutrition labels shift peoples’ attention to healthy foods and exert more influence on their choices. Nutrition Research. 2020; 80:106–116. 32739728 10.1016/j.nutres.2020.06.013

[R46] Sepulveda P, Usher M, Davies N, Benson AA, Ortoleva P, De Martino B. Visual attention modulates the integration of goal-relevant evidence and not value. Elife. 2020; 9:e60705. 10.7554/eLife.60705 PMC7723413 33200982

[R47] Shadlen MN, Shohamy D. Decision making and sequential sampling from memory. Neuron. 2016; 90(5):927–939. 27253447 10.1016/j.neuron.2016.04.036 PMC4891701

[R48] Shevlin BR, Smith SM, Hausfeld J, Krajbich I. High-value decisions are fast and accurate, inconsistent with diminishing value sensitivity. Proceedings of the National Academy of Sciences. 2022; 119(6):e2101508119. 10.1073/pnas.2101508119 PMC8832986 35105801

[R49] Shimojo S, Simion C, Shimojo E, Scheier C. Gaze bias both reflects and influences preference. Nature neuroscience. 2003; 6(12):1317–1322. 14608360 10.1038/nn1150

[R50] Smith SM, Krajbich I. Attention and choice across domains. Journal of Experimental Psychology: General. 2018; 147(12):1810. 30247061 10.1037/xge0000482

[R51] Smith SM, Krajbich I. Gaze amplifies value in decision making. Psychological science. 2019; 30(1):116–128. 30526339 10.1177/0956797618810521

[R52] Steinemann NA, Stine GM, Trautmann EM, Zylberberg A, Wolpert DM, Shadlen MN. Direct observation of the neural computations underlying a single decision. bioRxiv. 2022; p. 2022–05. 10.7554/eLife.90859 PMC11488853 39422555

[R53] Störmer VS, Alvarez GA. Attention alters perceived attractiveness. Psychological Science. 2016; 27(4):563–571. 26966228 10.1177/0956797616630964

[R54] Sullivan N, Hutcherson C, Harris A, Rangel A. Dietary self-control is related to the speed with which attributes of healthfulness and tastiness are processed. Psychological science. 2015; 26(2):122–134. 25515527 10.1177/0956797614559543 PMC4372728

[R55] Summerfield C, Tsetsos K. Do humans make good decisions? Trends in cognitive sciences. 2015; 19(1):27–34. 25488076 10.1016/j.tics.2014.11.005 PMC4286584

[R56] Suzuki S, Cross L, O’Doherty JP. Elucidating the underlying components of food valuation in the human orbitofrontal cortex. Nature neuroscience. 2017; 20(12):1780–1786. 29184201 10.1038/s41593-017-0008-x PMC6214455

[R57] Tavares G, Perona P, Rangel A. The attentional drift diffusion model of simple perceptual decision-making. Frontiers in neuroscience. 2017; 11:468. 28894413 10.3389/fnins.2017.00468 PMC5573732

[R58] Thomas AW, Molter F, Krajbich I. Uncovering the computational mechanisms underlying many-alternative choice. Elife. 2021; 10:e57012. 10.7554/eLife.57012 PMC8025657 33821787

[R59] Thomas AW, Molter F, Krajbich I, Heekeren HR, Mohr PN. Gaze bias differences capture individual choice behaviour. Nature Human Behaviour. 2019; 3(6):625–635. 10.1038/s41562-019-0584-8 30988476

[R60] Tolhurst D, Movshon JA, Thompson I. The dependence of response amplitude and variance of cat visual cortical neurones on stimulus contrast. Experimental brain research. 1981; 41:414–419. 7215502 10.1007/BF00238900

[R61] Trueblood JS, Brown SD, Heathcote A. The multiattribute linear ballistic accumulator model of context effects in multialternative choice. Psychological review. 2014; 121(2):179. 24730597 10.1037/a0036137

[R62] Tversky A. Elimination by aspects: A theory of choice. Psychological review. 1972; 79(4):281.

[R63] Usher M, McClelland JL. The time course of perceptual choice: the leaky, competing accumulator model. Psychological review. 2001; 108(3):550. 11488378 10.1037/0033-295x.108.3.550

[R64] Usher M, McClelland JL. Loss aversion and inhibition in dynamical models of multialternative choice. Psychological review. 2004; 111(3):757. 15250782 10.1037/0033-295X.111.3.757

[R65] Vickers D. Decision processes in visual perception. Academic Press; 1979.

[R66] Wang XJ. Probabilistic decision making by slow reverberation in cortical circuits. Neuron. 2002; 36(5):955–968. 12467598 10.1016/s0896-6273(02)01092-9

[R67] Westbrook A, Van Den Bosch R, Määttä J, Hofmans L, Papadopetraki D, Cools R, Frank M. Dopamine promotes cognitive effort by biasing the benefits versus costs of cognitive work. Science. 2020; 367(6484):1362–1366. 32193325 10.1126/science.aaz5891 PMC7430502

[R68] Yang X, Krajbich I. A dynamic computational model of gaze and choice in multi-attribute decisions. Psychological Review. 2023; 130(1):52. 35025570 10.1037/rev0000350

[R69] Zajonc RB. Attitudinal effects of mere exposure. Journal of personality and social psychology. 1968; 9(2p2):1.

[R70] Zylberberg A, Bakkour A, Shohamy D, Shadlen MN. Value construction through sequential sampling explains serial dependencies in decision making. Elife. 2024; 13:RP96997. 10.7554/eLife.96997 PMC11630821 39656196

[R71] Zylberberg A, Fetsch CR, Shadlen MN. The influence of evidence volatility on choice, reaction time and confidence in a perceptual decision. Elife. 2016; 5:e17688. 10.7554/eLife.17688 PMC5083065 27787198

