# Supplementary material for: Behavioral Signatures of Post-Decisional Attention in Preferential Choice
Source: bioRxiv. 2026 Jan 12:2026.01.10.698805. Preprint. [Version 1] doi: 10.64898/2026.01.10.698805 (PMC12871329; doi:10.64898/2026.01.10.698805)
Supplement: Supplement 1 [file NIHPP2026.01.10.698805v1-supplement-1.pdf]

bioRxiv preprint doi: https://doi.org/10.64898/2026.01.10.698805; this version posted January 12, 2026. The copyright holder for this preprint (which was not certified by peer review) is the author/funder, who has granted bioRxiv a license to display the preprint in perpetuity. It is made available under aCC-BY 4.0 International license.

823 # Supplemental information

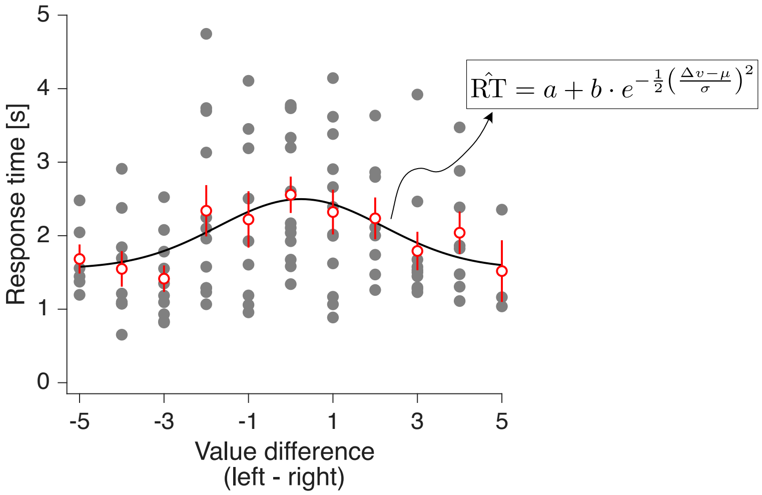

Figure S1. Removing the contribution of Δr from response times
Illustration of the method used to remove the contribution of Δr from the RT. The gray markers indicate the RT for each trial of a representative participant. The abscissa are the values of Δr for the corresponding trial. These data points were fitted with the bell-shaped function shown in the figure, with parameters a, b, μ and σ. The best-fitting function captures the general trend in the data, as can be seen by comparing the model fits (black solid line) with the average response time per value of Δr (red, mean plus s.e.m.). To compute the RT residuals, we subtract from each trial the value of $\hat{RT}$ corresponding to the corresponding value of Δr. Fits were performed independently for each participant.

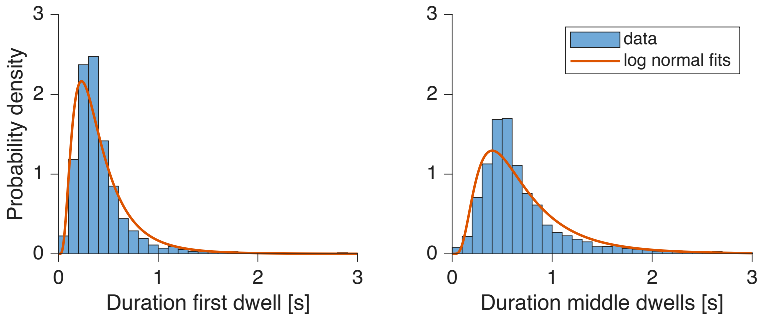

Figure S2. Fits of the duration of the dwells
Distribution of the durations of the first dwell (left) and middle dwells (right). Middle dwells include all dwells except the first and last. The durations were fitted with a log-normal distribution (red), independently for the first and middle dwells. The best-fitting log-normal parameters were used to simulate the *aDDM* and *gDDM*. On each trial, the first dwell is sampled from the corresponding distribution, and the subsequent dwell durations up to the bound crossing are sampled from the distribution of middle dwells.

bioRxiv preprint doi: https://doi.org/10.64898/2026.01.10.698805; this version posted January 12, 2026. The copyright holder for this preprint (which was not certified by peer review) is the author/funder, who has granted bioRxiv a license to display the preprint in perpetuity. It is made available under aCC-BY 4.0 International license.

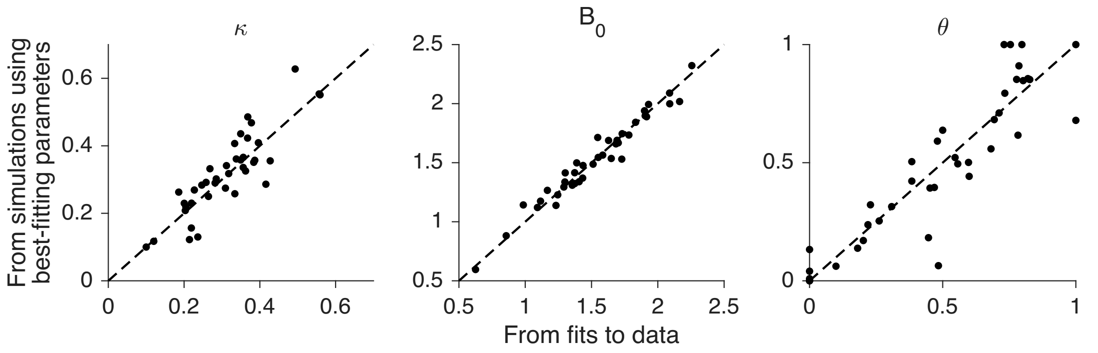

Figure S3. Parameter recovery for the *aDDM*
For each participant, we simulated choices and decision times using their individually best-fitting model parameters, with the same number of trials as in the original experiment. Each panel shows, for a given model parameter, the value estimated from the empirical data (x-axis) plotted against the corresponding value recovered from the simulated data (y-axis). Each point represents one participant (N=39).

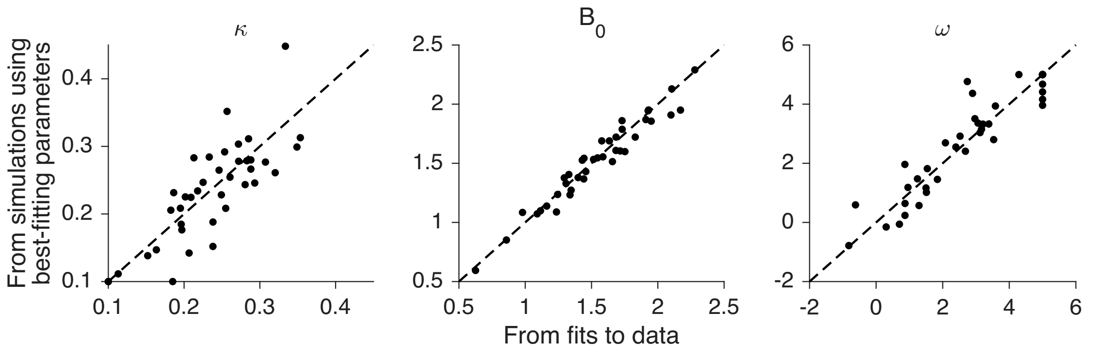

Figure S4. Parameter recovery for the *aDDM* variant with additive attention
Same as Fig. S3, for the model in which the effect of attention on choice is additive rather than multiplicative.

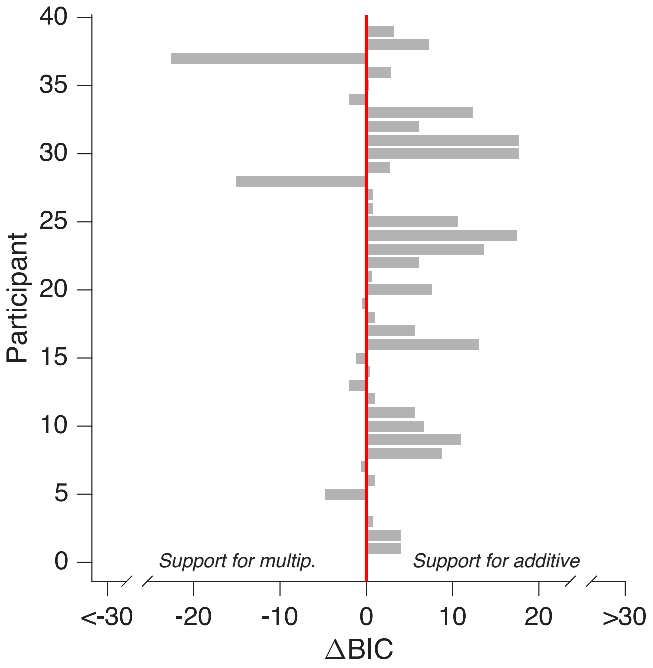

Figure S5. Relative goodness-of-fit of the additive and multiplicative models
Difference in Bayesian Information Criterion (ΔBIC) between models with multiplicative and additive attention effects. Positive values indicate support for the additive model. Overall, both models achieved comparable goodness-of-fit.

bioRxiv preprint doi: https://doi.org/10.64898/2026.01.10.698805; this version posted January 12, 2026. The copyright holder for this preprint (which was not certified by peer review) is the author/funder, who has granted bioRxiv a license to display the preprint in perpetuity. It is made available under aCC-BY 4.0 International license.

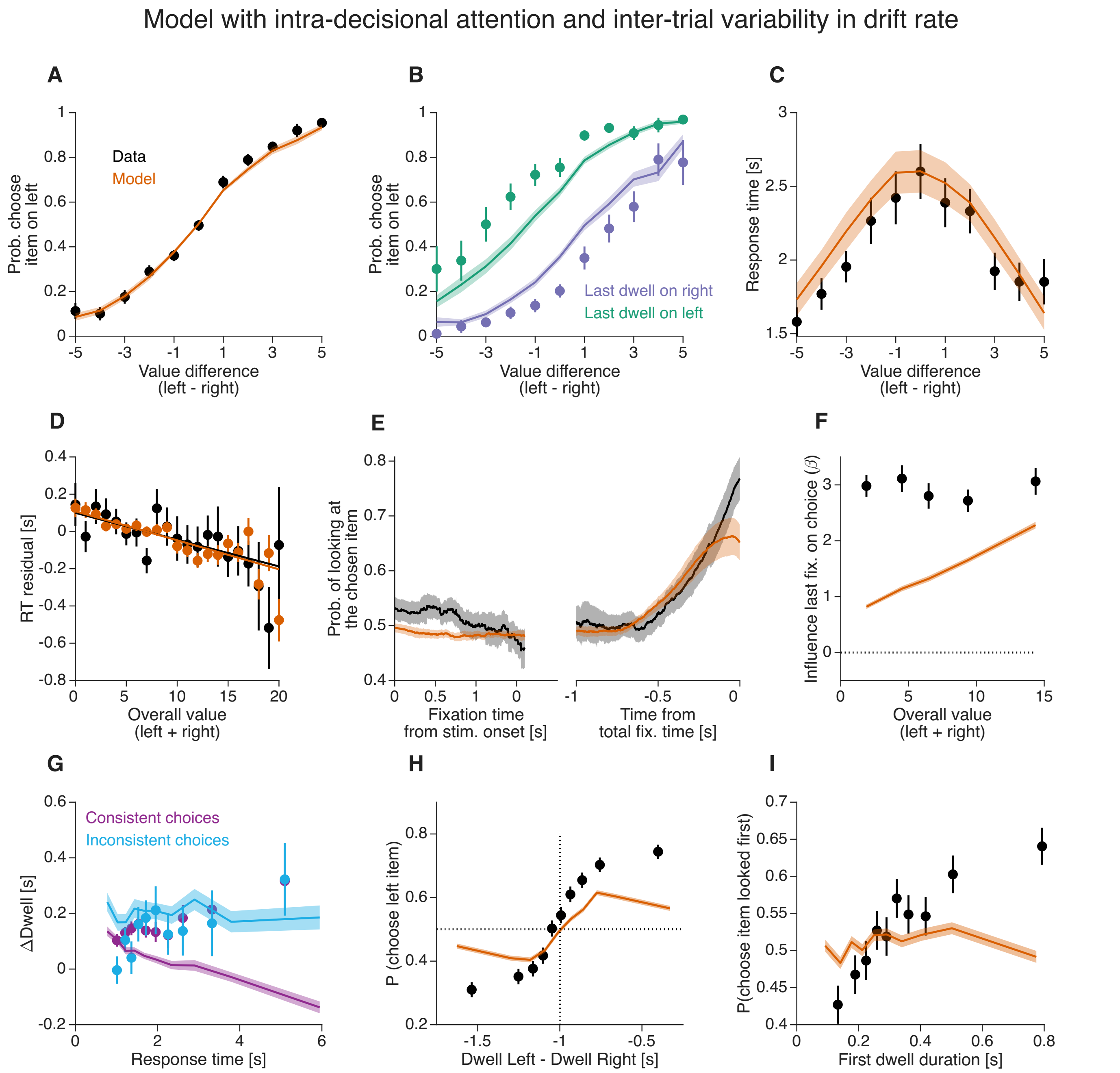

Figure S6. Model with intra-decisional attentional effects and inter-trial variability in the drift-rate
Same as Fig. 8 but including inter-trial variability in the drift-rate across trials. Same conventions as in Fig. 5.

bioRxiv preprint doi: https://doi.org/10.64898/2026.01.10.698805; this version posted January 12, 2026. The copyright holder for this preprint (which was not certified by peer review) is the author/funder, who has granted bioRxiv a license to display the preprint in perpetuity. It is made available under aCC-BY 4.0 International license.

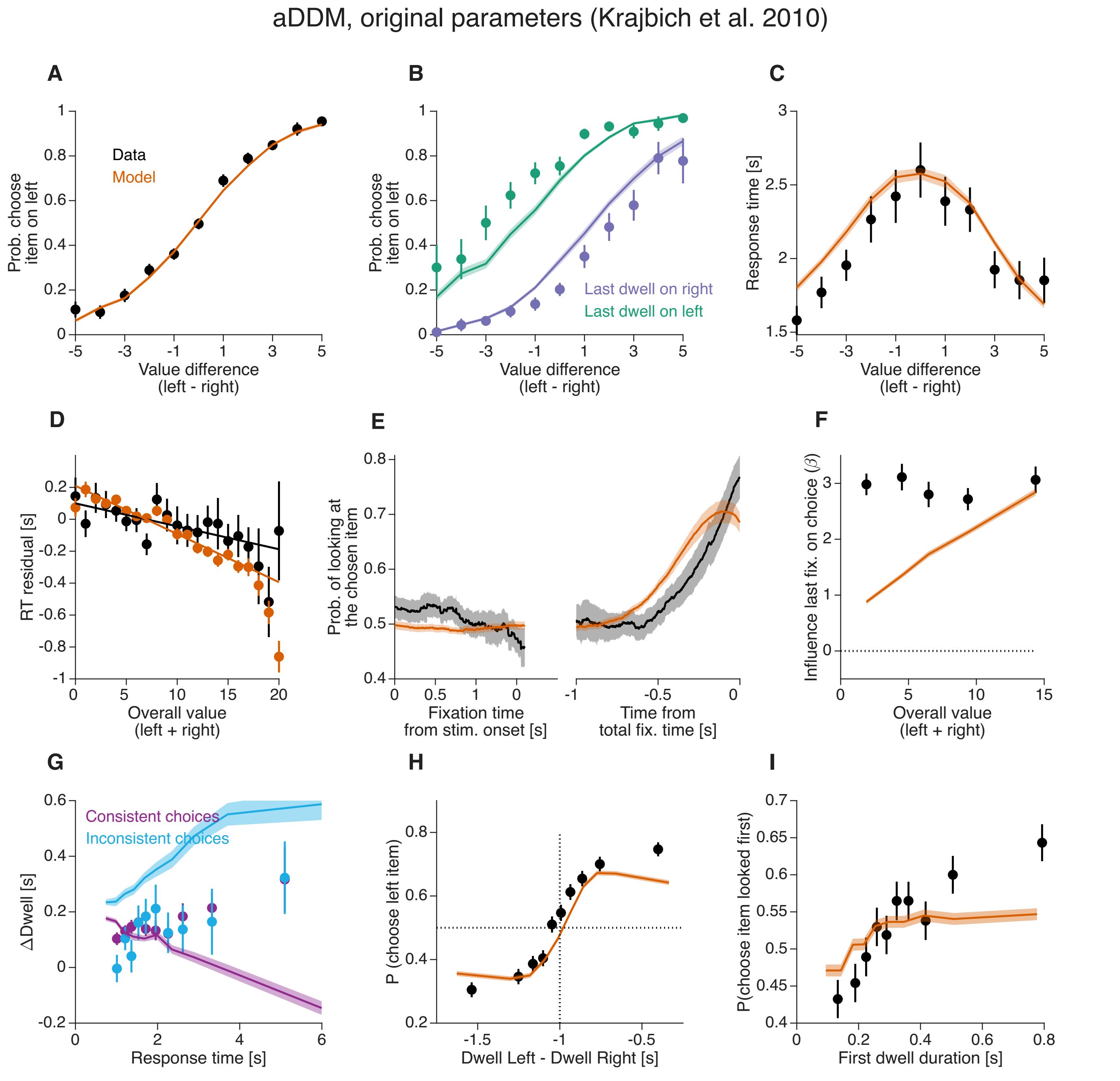

Figure S7. *aDDM* **model**

Model simulations were generated using the *aDDM* with the best-fitting parameters reported by Krajbich et al. (2010) and Smith and Krajbich (2019). This version of the model assumes constant (i.e., flat) decision bounds and no inter-trial variability in drift rate. Unlike the models presented in the main text, it was fit by Krajbich et al. (2010) to data pooled across participants. Same conventions as in Fig. 5.

bioRxiv preprint doi: https://doi.org/10.64898/2026.01.10.698805; this version posted January 12, 2026. The copyright holder for this preprint (which was not certified by peer review) is the author/funder, who has granted bioRxiv a license to display the preprint in perpetuity. It is made available under aCC-BY 4.0 International license.

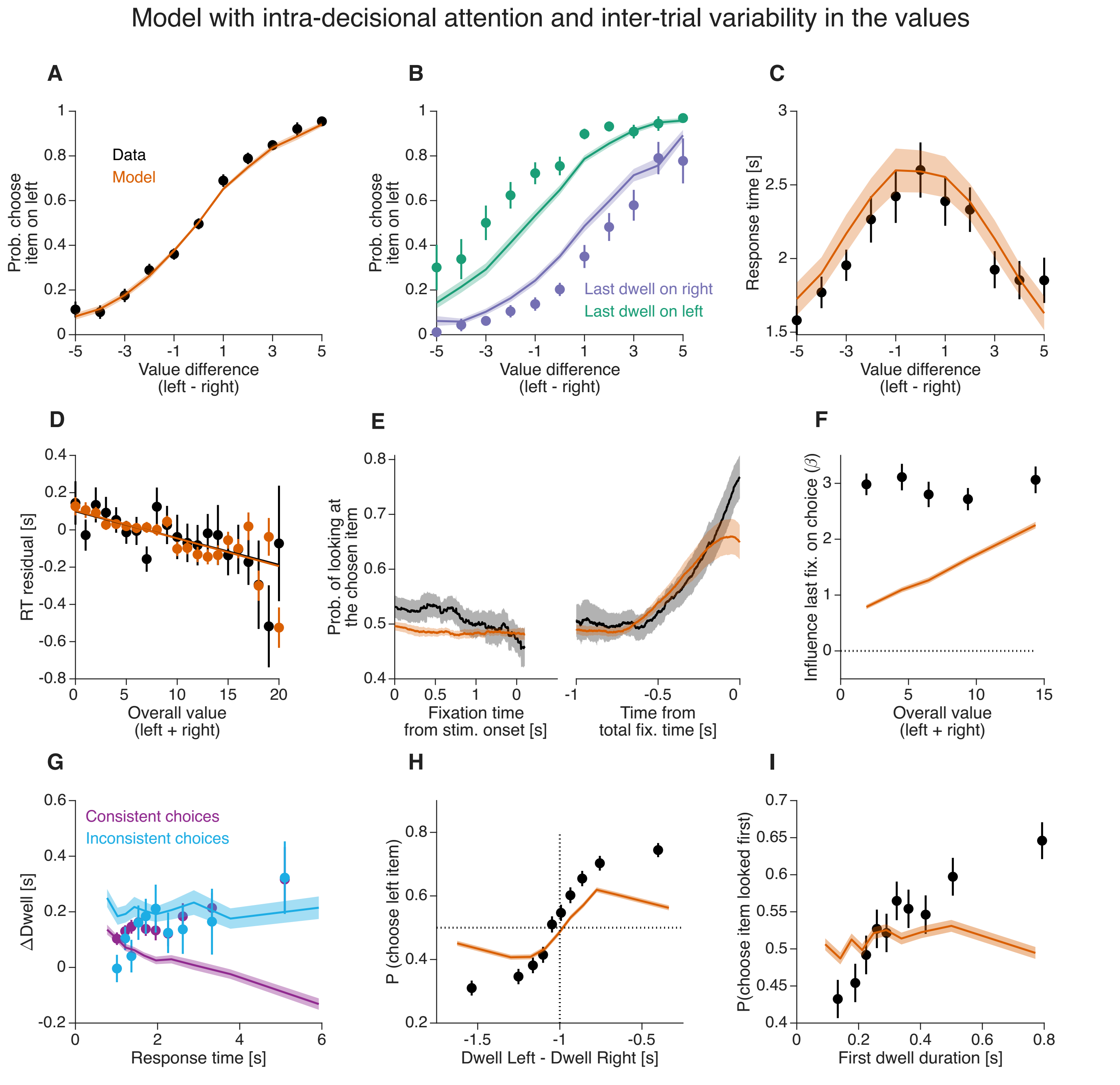

Figure S8. Model with intra- and post-decisional attention, and inter-trial variability in the items' value
Unlike the model illustrated in Fig. 11, here the items' values—instead of the drift rates—are corrupted with additive Gaussian noise. This noise is fixed within each trial but varies randomly across trials. Model parameters were fit to individual participants' choice, RT, and fixation data. Same conventions as in Fig. 5.

bioRxiv preprint doi: https://doi.org/10.64898/2026.01.10.698805; this version posted January 12, 2026. The copyright holder for this preprint (which was not certified by peer review) is the author/funder, who has granted bioRxiv a license to display the preprint in perpetuity. It is made available under aCC-BY 4.0 International license.

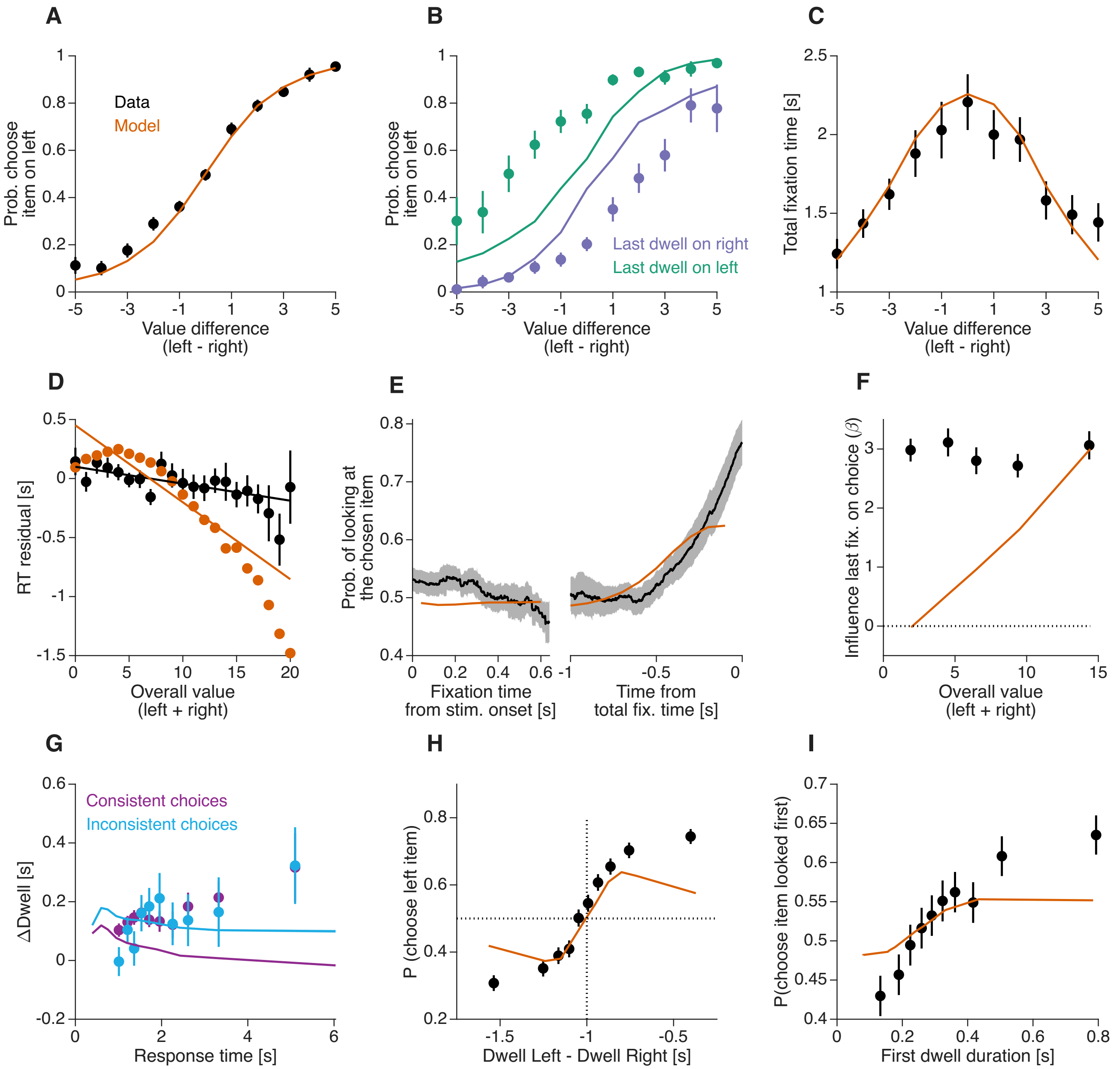

Figure S9. Model of Callaway et al. (2021)
Model simulations were obtained from the approximately optimal model of Callaway et al. (2021). Same conventions as in Fig. 5.

bioRxiv preprint doi: https://doi.org/10.64898/2026.01.10.698805; this version posted January 12, 2026. The copyright holder for this preprint (which was not certified by peer review) is the author/funder, who has granted bioRxiv a license to display the preprint in perpetuity. It is made available under aCC-BY 4.0 International license.

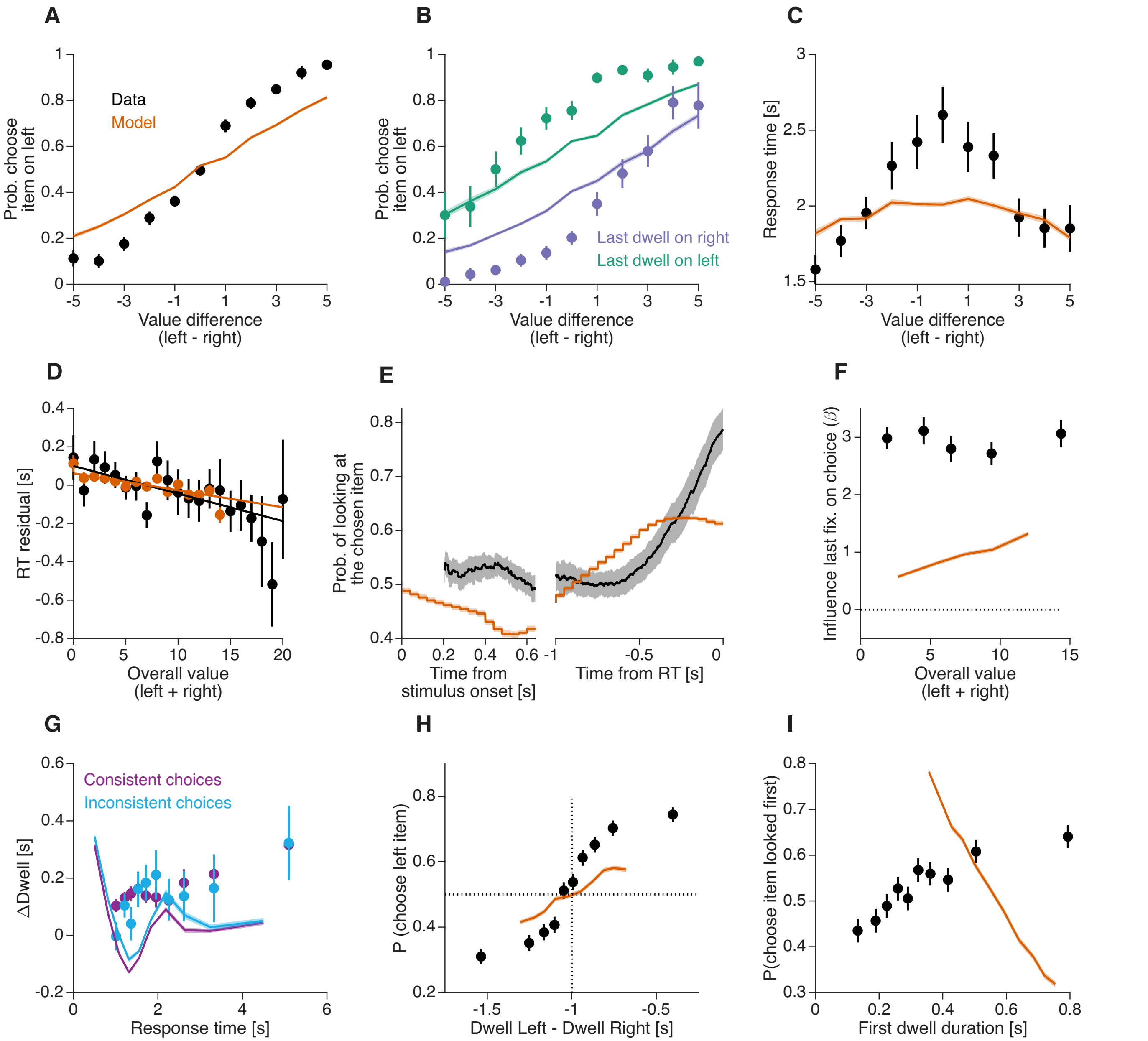

Figure S10. Model of Jang et al. (2021)
Model simulations were generated with the optimal model of Jang et al. (2021). Same conventions as in Fig. 5.

bioRxiv preprint doi: https://doi.org/10.64898/2026.01.10.698805; this version posted January 12, 2026. The copyright holder for this preprint (which was not certified by peer review) is the author/funder, who has granted bioRxiv a license to display the preprint in perpetuity. It is made available under aCC-BY 4.0 International license.

Subject	κ	μ_{nd}	σ_{nd}	B	γ
1	0.369	0.536	0.018	1.293	0.000
2	0.076	0.671	0.091	1.311	0.067
3	0.187	0.600	0.144	2.084	0.000
4	0.216	1.219	0.235	0.999	0.141
5	0.383	0.544	0.010	2.004	0.171
6	0.244	0.886	0.140	0.613	0.000
7	0.197	0.536	0.035	2.116	0.055
8	0.617	1.090	0.143	1.612	0.247
9	0.551	0.702	0.044	1.254	0.137
10	0.292	0.706	0.142	1.483	0.038
11	0.353	0.730	0.012	1.467	0.000
12	0.243	0.525	0.010	1.547	0.000
13	0.242	0.646	0.013	1.707	0.021
14	0.332	0.649	0.011	1.201	0.000
15	0.058	0.598	0.010	2.184	0.233
16	0.335	0.738	0.143	1.065	0.000
17	0.224	0.774	0.010	2.150	0.024
18	0.198	1.498	0.338	1.810	0.021
19	0.459	0.551	0.010	2.487	0.212
20	0.192	0.778	0.163	1.673	0.045
21	0.349	0.523	0.010	2.079	0.296
22	0.425	1.255	0.195	1.298	0.009
23	0.535	0.879	0.108	0.994	0.180
24	0.314	1.225	0.222	1.024	0.061
25	0.348	0.973	0.129	0.888	0.025
26	0.203	0.626	0.017	1.243	0.000
27	0.468	0.538	0.047	0.862	0.145
28	0.057	1.071	0.010	1.150	0.002
29	0.258	0.654	0.023	1.916	0.047
30	0.291	0.680	0.182	1.448	0.000
31	0.363	0.540	0.010	1.438	0.176
32	0.340	0.679	0.017	1.622	0.076
33	0.450	0.880	0.163	0.702	0.000
34	0.439	0.944	0.145	1.110	0.000
35	0.418	0.615	0.086	1.116	0.320
36	0.281	0.735	0.178	1.057	0.095
37	0.066	0.859	0.156	0.866	0.000
38	0.268	0.722	0.014	1.668	0.004
39	0.259	0.662	0.034	1.336	0.007

Table S1. Best-fitting parameter values for the *PDG* model.

bioRxiv preprint doi: https://doi.org/10.64898/2026.01.10.698805; this version posted January 12, 2026. The copyright holder for this preprint (which was not certified by peer review) is the author/funder, who has granted bioRxiv a license to display the preprint in perpetuity. It is made available under aCC-BY 4.0 International license.

Subject	κ	B	θ
1	0.368	1.433	0.802
2	0.120	1.291	0.262
3	0.219	2.164	0.797
4	0.214	0.625	0.731
5	0.282	1.355	0.500
6	0.186	1.115	1.000
7	0.203	1.930	0.452
8	0.384	1.702	0.202
9	0.427	1.374	0.597
10	0.356	1.731	0.308
11	0.356	1.649	0.786
12	0.227	0.857	1.000
13	0.247	1.550	0.820
14	0.309	1.245	0.827
15	0.100	1.584	0.484
16	0.349	1.388	0.681
17	0.268	2.257	0.546
18	0.205	1.692	0.778
19	0.338	1.832	0.694
20	0.264	1.684	0.219
21	0.258	1.092	0.469
22	0.362	2.090	0.712
23	0.312	1.299	0.733
24	0.416	1.728	0.000
25	0.349	1.547	0.181
26	0.220	1.512	0.783
27	0.334	0.985	0.447
28	0.396	1.782	0.000
29	0.285	1.915	0.384
30	0.387	1.905	0.000
31	0.334	1.407	0.480
32	0.556	1.900	0.000
33	0.368	1.302	0.599
34	0.559	1.629	0.229
35	0.493	1.167	0.099
36	0.236	1.232	0.754
37	0.200	1.373	0.000
38	0.318	2.089	0.557
39	0.378	1.436	0.384

Table S2. Best-fitting parameter values for the *aDDM*.

bioRxiv preprint doi: https://doi.org/10.64898/2026.01.10.698805; this version posted January 12, 2026. The copyright holder for this preprint (which was not certified by peer review) is the author/funder, who has granted bioRxiv a license to display the preprint in perpetuity. It is made available under aCC-BY 4.0 International license.

Subject	κ	B	ω
1	0.334	1.442	1.511
2	0.100	1.294	5.000
3	0.197	2.171	0.700
4	0.185	0.625	0.866
5	0.218	1.338	3.531
6	0.186	1.115	-0.820
7	0.152	1.927	3.584
8	0.285	1.730	5.000
9	0.349	1.399	2.522
10	0.261	1.731	5.000
11	0.320	1.658	1.499
12	0.238	0.858	-0.620
13	0.225	1.545	0.302
14	0.281	1.245	0.869
15	0.100	1.586	0.864
16	0.293	1.430	2.897
17	0.213	2.279	3.123
18	0.183	1.685	1.289
19	0.288	1.830	0.957
20	0.163	1.684	5.000
21	0.195	1.090	4.289
22	0.307	2.099	2.395
23	0.272	1.346	2.682
24	0.249	1.752	5.000
25	0.233	1.577	5.000
26	0.196	1.515	1.241
27	0.238	0.980	2.740
28	0.209	1.716	5.000
29	0.202	1.930	3.166
30	0.272	1.950	5.000
31	0.255	1.457	2.967
32	0.288	1.910	3.207
33	0.283	1.329	3.061
34	0.353	1.635	2.081
35	0.254	1.162	5.000
36	0.207	1.236	1.538
37	0.113	1.308	5.000
38	0.246	2.105	3.384
39	0.257	1.443	1.841

Table S3. Best-fitting parameter values for the model with additive intra-decision attention.

bioRxiv preprint doi: https://doi.org/10.64898/2026.01.10.698805; this version posted January 12, 2026. The copyright holder for this preprint (which was not certified by peer review) is the author/funder, who has granted bioRxiv a license to display the preprint in perpetuity. It is made available under aCC-BY 4.0 International license.

Subject	κ	B	θ	σ_{drift}
1	0.369	1.435	0.803	0.000
2	0.158	1.495	0.405	0.804
3	0.223	2.179	0.799	0.095
4	0.213	0.625	0.727	0.000
5	0.464	1.804	0.576	1.205
6	0.335	1.762	1.000	1.494
7	0.263	2.172	0.532	0.490
8	0.570	2.514	0.224	1.125
9	0.430	1.377	0.596	0.091
10	0.578	2.463	0.415	1.062
11	0.494	2.041	0.791	0.808
12	0.227	0.855	1.000	0.007
13	0.495	2.075	0.877	1.050
14	0.387	1.356	0.868	0.621
15	0.100	1.788	0.634	0.681
16	0.373	1.439	0.678	0.336
17	0.871	5.911	0.590	2.128
18	0.204	1.690	0.778	0.000
19	0.861	3.409	0.669	1.543
20	0.385	2.144	0.293	0.816
21	0.258	1.090	0.468	0.007
22	1.160	6.722	0.721	2.582
23	0.381	1.553	0.772	0.822
24	0.854	3.662	0.000	1.740
25	0.466	2.039	0.235	0.927
26	0.422	2.157	0.803	1.178
27	0.378	1.101	0.477	0.815
28	0.686	2.889	0.000	1.361
29	0.357	2.164	0.443	0.522
30	0.551	2.482	0.000	0.886
31	0.502	1.822	0.563	1.000
32	0.839	2.622	0.114	0.884
33	0.512	1.763	0.641	1.057
34	0.708	1.968	0.301	0.688
35	0.856	1.827	0.260	1.555
36	0.309	1.410	0.778	0.738
37	0.384	2.248	0.000	1.460
38	0.445	2.581	0.604	0.637
39	0.628	1.887	0.447	1.062

Table S4. Best-fitting parameter values for the *aDDM* with inter-trial drift-rate variability.
